# Mechanisms of unique chaotropic resistance and biocalcification in a halophilic *Virgibacillus* isolated from ephemeral microbial mats of the calcium-rich Don Juan pond in Antarctica

**DOI:** 10.3389/fmicb.2026.1980805

**Published:** 2026-10-28

**Authors:** Laura Marturano, Violetta La Cono, Francesco Smedile, Gina La Spada, Francesca Crisafi, Elena A. Selivanova, Marina E. Ignatenko, Manasi Mudaliyar, Debnath Ghosal, Enzo Messina, Alfonso Modica, Oleg N. Reva, Sergey N. Gavrilov, Michail M. Yakimov

**Affiliations:** 1 Institute of Polar Sciences, ISP-CNR, Messina, Italy; 2 Orenburg Federal Research Center, Institute for Cellular and Intracellular Symbiosis, Ural Branch of RAS, Orenburg, Russia; 3 Department of Biochemistry and Pharmacology, Bio21 Molecular Science and Biotechnology Institute, The University of Melbourne, Melbourne, VIC, Australia; 4 ARC Centre for Cryo-electron Microscopy of Membrane Proteins, Bio21 Molecular Science and Biotechnology Institute, University of Melbourne, Parkville, VIC, Australia; 5 Institute for Marine Biological Resources and Biotechnology, IRBIM-CNR, Messina, Italy; 6 Eni Rewind EE&MD/SELAB, Priolo Gargallo (SR), Italy; 7 Department of Biochemistry, Genetics and Microbiology, Centre for Bioinformatics and Computational Biology, University of Pretoria, Pretoria, South Africa; 8 Winogradsky Institute of Microbiology, Research Centre of Biotechnology RAS, Moscow, Russia

**Keywords:** astrobiology, athalassohaline and chaotropic brine lakes, biocalcification, carbonic anhydrase, polyextremophiles, cryo-ET, transcriptome analysis, *Virgibacillus*

## Abstract

**Introduction:**

Don Juan Pond (DJP), located in the McMurdo Dry Valleys of Antarctica, is one of the most extreme cold aquatic environments on our planet and most similar to Mars due to its CaCl₂-rich brines, low water activity, exceptional chaotropicity, average annual subzero temperatures and intense solar radiation during the Antarctic summer. Although the central DJP brine is considered anathema to life, the surrounding basin seasonally hosts temporary streams, ponds, and, very rarely, ephemeral microbial mats.

**Methods:**

In this study, we investigated the diversity of these microbial mats and attempted to cultivate Ca^2+^-tolerant bacteria and analyze the phenotypic and molecular characteristics of the most promising isolates. To understand the molecular mechanisms underlying their adaptive strategy, further analysis of their genomic potential, adaptive epigenetic transformations and differential transcriptomics was conducted. Predicted functions were verified through physiological experiments.

**Results:**

The most calcium-tolerant isolate, *Virgibacillus flavescens* DJP025-4, was studied in detail, including its adaptive physiology and, in particular, its remarkable tolerance to varying calcium concentrations, unmatched by other prokaryotes. DJP025-4 cells were able to survive and remain cultivable after 3 months of exposure to 3.0 M Ca^2+^, corresponding to a water activity of approximately 0.63 and total chaotropicity value of +276.6 kJ kg^−1^, conditions well below the current limit for life survival equivalent. Transcriptome analysis revealed that the primary metabolic response of strain DJP025-4 to Ca^2+^ stress includes enzymatically induced urease-independent CaCO_3_ precipitation, enhanced uptake of external compatible solutes, and activation of cell surface structures biosynthesis, including peptidoglycan metabolism, biofilm matrix formation, and cellular appendage assembly, which finally increase the cell surface area and the number of Ca^2 +^ precipitation centers on it. Whole-genome analysis revealed a high degree of genetic plasticity in this bacterium, involving natural transformation with genetic material acquired from the bacterial community and adaptive epigenetic mechanisms of gene regulation.

**Discussion:**

Among the key findings, it is worth noting that, in addition to developing remarkable adaptation mechanisms to withstand and thrive in a harsh, calcium-rich environment, strain DJP025-4 appears to remain dependent on the presence of external compatible solutes. We hypothesize that within the DJP stream ecosystem, it is able to survive at high Ca^2+^ concentrations by utilizing trace amounts of such substances produced by other organisms, specifically, moderate halophiles inhabiting surface microbial mats in lower-salinity zones, which release dissolved organic matter and osmolytes into the environment.

## Introduction

The study of polyextremophile organisms thriving in the harshest conditions on our planet, resembling extraterrestrial environments, provides us with invaluable information about the potential signatures of life beyond Earth, which is almost certainly widespread in the Universe, as well as helps to understand the diversity of adaptation mechanisms inherent in living forms of the Earth. As is known, several basic conditions are necessary for the existence of life: (i) the presence of liquid water with sufficient thermodynamic bioavailability, termed water activity (a_w_); (ii) the presence of an energy source(s), provided by suitable pairs of electron donors and acceptors; and (iii) a suitable chemical and physical environment, including appropriate temperature, salinity, moderate radiation, and moderate chaotropicity (i.e., entropic disruption of the network of hydrogen bonds between water molecules, leading to the disruption of the integrity of biomacromolecules). Our understanding of the limits of life is constantly evolving based on extensive research in this area and demonstrates the incredible adaptability of microorganisms to metabolic activity at the edge of life. The a_w_ limit for the most extremely halophilic bacteria and archaea was long believed to be 0.755, and ionic and organic solutes were thought to impose both qualitatively and quantitatively different biophysical constraints on microbial systems. However, recent studies have shown that biotic activity, i.e., cell division, can occurs at a_w_ values close to 0.600 for halophilic bacteria and archaea in some saline milieu (Stevenson et al., 2015), and to 0.585 for an ascomycete fungus in glycerol-supplemented media (Stevenson et al., 2017).

Exploration of the Martian surface using rovers and orbital technologies has revealed the presence of repeating linear structures on slopes that are thought to represent seasonal brines or hydrated minerals rich in Mg^2+^/Ca^2+^, indicating the existence of athalassohaline brine paleolakes that formed periodically on ancient Mars (Burt and Knauth, 2003; Dickson et al., 2013; de Vera et al., 2019). These lakes were widespread in time and space and were likely 10–100 times saltier than terrestrial seawater (Tosca et al., 2008). Due to their potential for habitability and the preservation of biosignatures in sediments formed in quiescent conditions, paleolakes are considered priority targets for astrobiological studies of Mars (Fox-Powell et al., 2016). Interestingly, similar athalassohaline hydrological formations, where such divalent cations, rather than sodium, are strictly dominant, are also known on our planet. High-calcium environments are widespread in karst caves—specifically, in the areas where calcitic and gypsic speleothems form (Banks et al., 2010). Microorganisms must cope with the toxic effects of Ca^2+^ ions by employing various strategies, notably the deposition and immobilization of calcium outside the cell through biocalcification processes (Tisato et al., 2015). However, cave aquatic microenvironments are characterized by normal water activity and do not exert the kind of evolutionary pressure on calcifying microorganisms that would necessitate the development of mechanisms for high salt tolerance or resistance to chaotropic stress. The most prominent examples of extremely chaotropic environments are the Mediterranean deep-sea hypersaline lakes *Discovery*, *Hephaestus*, and *Kryos*, where brines are saturated with MgCl_2_ (~ 5.0 M). By studying them, it was demonstrated that Mg^2+^ is a strong chaotropic and osmotic stressor, and such hydrochemical conditions are unfavorable for life processes, that is, the lakes themselves are anathema to life (Hallsworth et al., 2007; Yakimov et al., 2015; La Cono et al., 2019; Polonia et al., 2026).

Alongside the Mg^2+^-rich deep-sea brine lakes and subsurface calcium-rich environments, there is also a unique body of water on Earth in which calcium chloride is absolutely dominant—Don Juan Pond (DJP), located at altitude of 162 m above sea level in the South Fork of Wright Valley, McMurdo Dry Valleys, Victoria Land, Antarctica (77°33′S, 162°11′E). DJP is a small, shallow (average depth 15–25 cm and size approximately 200 × 700 m) pond with an extraordinary salinity (some studies report values exceeding 600 g l^−1^ and Ca^2+^ concentrations up to 6.0 M), which maintains its liquid state even during austral winter—the eutectic point for the DJP brine is estimated to be −52 °C (Marion et al., 2003). Since it was discovered in 1961, DJP has intrigued biologists searching for the most extreme environments on Earth that support life. Extreme salinity of DJP causes the a_w_ values to drop to 0.41–0.45, well below the theoretical limit for the replication and metabolism of all known organisms. Moreover, the extreme concentration of Ca^2+^ ions exerts a strong chaotropic effect on biological systems, destabilizing biomacromolecules by disrupting hydrogen-bonding networks, destroying protein hydration shells, and denaturing membrane lipid bilayers. All these theoretical arguments appear to indicate that the internal environment of DJP is devoid of life.

Nevertheless, a limited number of publications spanning more than half a century indicate that microbial communities were occasionally detected on the western margins of the DJP, particularly in areas where glacial meltwater entered the DJP basin (Meyer et al., 1962a; Meyer et al., 1962b; Siegel et al., 1979; Shaffer et al., 2023). Recent geochemical studies have shown that the presence of CaCl_2_-rich brine in the DJP basin is explained by its local upwelling from a regional groundwater system, in which evaporated DJP brines return to the subsurface throughout the year (Dickson et al., 2013; Toner et al., 2017). Due to fluctuations in groundwater flow, particularly in marginal zones and in areas of seasonal meltwater inflow, CaCl_2_ concentrations vary from year to year. This zone may occasionally exhibit lower salinity/chaotropicity and thus, may represent an ecological niche populated by Ca^2+^-tolerant microbiota, likely formed by eolian deposition. However, in years with low water levels, these allochthonous microbial communities die out or become dormant. Thus, according to the current understanding of microbial life at DJP, there is no autochthonous microbial community, but only a transient marginal community resilient to multiple stresses caused by UV radiation, desiccation, low water activity, and extreme chaotropicity. In the austral summer of 1978–79, an extensive, irregular film or mat-like structure, 2–5 mm thick, was observed, extending for 500–600 m^2^ across most of the western part of the Don Juan Pond salt marshes, at the inlet of meltwater from a nearby glacier. A similar biological formation reappeared only 40 years later, and the four cultured isolates were described as the UV-resistant red-pigmented *Betaproteobacteria Massilia frigida* strain DJPM01, *Alphaproteobacteria Loktanella* sp. DJP18 and two bacilli, *Bacillus* sp. DJP31 and *Virgibacillus* sp. DJP39 (Shaffer et al., 2023, 2026).

This paper presents the results of a biomolecular study of a sparse microbial community observed as bubble microbial mats on the southern periphery of DJP in November–December 2022, where CaCl_2_-rich subsurface brine mixed with meltwater from a nearby glacier forms a slow-flowing shallow stream with a calcium concentration gradient of 1.05–2.75 M from surface to bottom. Attempts to culture bacteria at calcium concentrations similar to those observed in natural conditions were also undertaken, resulting in the recovery of several isolates belonging to the phyla *Bacillota* and *Pseudomonadota*. The most calcium-tolerant isolate, *Virgibacillus flavescens* DJP025-4, was studied in more detail, including its adaptive physiology and, in particular, its remarkable tolerance to varying concentrations of calcium, chloride and perchlorate brines. To understand the molecular mechanisms underlying its adaptive strategy including biocalcification activity, we performed physiological experiments, microscopy, whole-genome sequencing and differential transcriptomics were combined to identify cellular and molecular mechanisms underlying its adaptation to extreme calcium concentrations, including biocalcification and stress-responsive metabolic processes. In addition, we investigated DNA methylation as a potential epigenetic component of environmental adaptation. In bacteria, condition-dependent changes in DNA methylation may provide a reversible mechanism influencing gene regulation without changes in the underlying DNA sequence (Casadesús and Low, 2006; Sánchez-Romero and Casadesús, 2020; Smedile et al., 2025). We therefore compared genome-wide methylation patterns under normal and Ca-stress conditions to determine whether exposure to chaotropic stress is accompanied by changes in the epigenetic state of the genome.

## Experimental procedures

### Samples, enrichments, and growth conditions

Sampling of ephemeral microbial mats was conducted during the austral summer (November–December 2022) at the DJP site, located at an elevation of 162 m above sea level in the South Fork of Wright Valley, McMurdo Dry Valleys, Victoria Land, Antarctica (77°33′S, 162°11′E). All samples were collected under strict security precautions using clean access technology. This aspect was crucial to the study, as the DJP site is located within the Antarctic McMurdo Dry Valleys Specially Protected Area, which encompasses the largest ice-free region in Antarctica. Therefore, samplings were performed using sterile suites and sterile high boots for personnel. All equipment was sterilized at Mario Zucchelli Station before use, transported in closed sterile boxes, and samples were collected using sterile gloves. Microbial mat samples were collected in minimal quantities (2–10 g) using sterile polypropylene spatulas. Whenever possible, a portion of the overlying water was collected along with the topmost sediment layer to preserve the structure of the microbial mats. Samples were transferred to sterile 50 mL Falcon^®^ conical tubes, minimizing the disturbance of the microbial mat. After collection, all samples for microbiological work were stored at approximately 4 °C in the dark during fieldwork and transported to Mario Zucchelli Station and then to Italy for laboratory processing (June 2023). Samples (up to 200 mL) for determination of major ion concentrations were collected in dark-polyethylene (DPE) vials and stored at −4 °C temperature until chemical analysis, following protocols previously developed for the characterization of athalassohaline brines from deep-sea hypersaline lakes of the Mediterranean Sea (Hallsworth et al., 2007; Yakimov et al., 2015; La Cono et al., 2019). Given the extensive literature on the water activity measurements and the chaotropic properties of CaCl₂ solutions, all values were estimated theoretically based on published data and using the Pitzer equations and molal osmotic coefficient *φ*(CaCl_2_) (Teng and Lenzi, 1974; Staples and Nuttall, 1977; Rard and Clegg, 1997; Bui et al., 2003; Cray et al., 2013, 2015). Since the highly stratified brine from the DJP025 sampling point consists of a virtually pure calcium chloride solution with minimal admixture of other salts, we considered it reasonable to assume that the values determined in this manner would not differ significantly from the actual parameters.

Upon the samples arrived in Italy, cultivation attempts were made to proceed with them under conditions simulating the environment of the sampling site. For this, the liquid neutral nutrient medium MBSW70 + 1 M Ca^2+^ with the following composition was used (g l^−1^): 40.3 dehydrated commercial powder of standard Marine Broth 2216 (Millipore 76448), 70 NaCl, 5 KCl. The pH was adjusted to 7.4 by the addition of 1 M KOH. After sterilization (121 °C, 20 min) and cooling, the medium was supplemented with 111 g l^−1^ CaCl_2_ (corresponding to 1 M), 1 mL l^−1^ acidic trace metal solution and 1 mL l^−1^ vitamin mix (Pfennig and Trüper, 1992). For initial enrichments, the microbial mat inoculum (5% w/vol) was added to the MBSW70 + 1 M medium and incubated on a rotary shaker at 120 rpm in 120-mL serum vials with a total liquid volume of 50 mL, closed with cotton stopper, for 3 months at 12 °C. The strategy for isolating calcium-resistant bacteria consisted of several rounds of 10-fold dilution in fresh MBSW70 + 1 M medium (vol/vol), followed by two repeats of serial dilution-to-extinction up to 10^−7^ in the same medium. After turbidity appeared, 50 μL of the grown culture was diluted in 5 mL of liquid medium, and then 20 μL of the resulting dilution were plated on MBSW70 + 1 M agar plates (1.5% w/vol). The grown colonies of different size and morphologies were picked and inoculated into liquid MBSW70 medium (without addition of CaCl_2_) for further analyses.

### Screening of calcium-tolerant isolates and taxonomic identification

The obtained isolates were subjected to preliminary screening experiments for the capabilities of growth in MBSW70 + 1 M medium supplemented with CaCl_2_ up to 2.0 M (222 g l^−1^ in total) and monitored by the increased turbidity (measured as increase in optical density at OD 600 nm). As a result, four promising isolates were obtained, which were subjected to taxonomic identification by sequencing their 16S rRNA genes. Bacterial DNA was extracted using GenElute™ Bacterial Genomic DNA Kits, following manufacturer’s instructions. PCR amplification of the 16S rRNA genes was performed using 1 ng of the extracted genomic DNA as the template and the universal primers 27F (5′-AGAGTTTGATCCTGGCTCAG-3) and 1492R (5′-TACGGYTACCTTGTTAGCACTT-3′). Thermocycler conditions began with initial denaturation at 95 °C for 1 min, followed by 30 cycles at 95 °C for 30 s, annealing at 55 °C for 45 s, and extension at 72 °C for 1.5 min, and a final polymerization extension at 72 °C for 5 min. PCR amplification was verified by 1% agarose gel electrophoresis and those showing single positive amplicon were purified and then sent for Sanger sequencing at the Macrogen Europe Sequencing Centre.^1^ Further classification of 16S rRNA genes was performed with ARB 7.0 software (Ludwig et al., 2004) on SILVA SSU 138.2 database (Chuvochina et al., 2026).

High-throughput 16S rRNA amplicon sequencing of DJP025 microbial mats.

Next-generation sequencing was used to study the biodiversity of the DJP025 microbial mats as it is described in detail elsewhere (Smedile et al., 2024). Briefly, to analyze the prokaryotic community, the V3–V4 variable regions of the SSU rRNA gene were amplified using universal primers S-D-Bact-0341-b-S-17, 5′-CCTACGGGNGGCWGCAG-3′ and S-D-Bact-0785-a-A-21, 5′-GACTACHVGGGTATCTAATCC-3′, as described by Caporaso et al. (2012) and Klindworth et al. (2013). Although, these primers ensure sufficient coverage of both bacteria and archaea, additionally, the specific primer pair SSU1ArF + SSU520R (Bahram et al., 2019) was used to detect the potential presence of archaea in the study area. Amplicons were sequenced on an Illumina MiSeq platform by Macrogen Europe Sequencing Centre (see text footnote 1). Raw reads were processed according to Smedile et al. (2024) and imported in RStudio package and analyzed using the DADA2 package (v 1.26.0) (Callahan et al., 2016). A parametric error model, based on the convergence between the estimation of error rate and the inference of the sample composition, was applied to remove PCR biases. Then, paired-end reads were merged and the amplicon sequence variants (ASVs) table was constructed. After chimeric sequences were removed, taxonomy was assigned to ASVs using the naive Bayesian classifier method against the Silva Database v138.2.^2^,^3^ Alpha diversity analysis was performed using Phyloseq and Microbiome packages (McMurdie and Holmes, 2013; Shetty and Lahti, 2019).

### DJP025-4 genome sequencing, assembly, and annotation

The strain *Virgibacillus flavescevs* DJP025-4 was selected as one the most promising Ca-resilient strains because it had a remarkable ability to survive and grow at 2.0 M CaCl_2_ concentrations. Genome was sequenced at the Macrogen Europe Sequencing Centre (see text footnote 1) using the Illumina NovaSeq platform, and the 18,905,936 paired-end raw reads generated in FASTq format were subjected to quality assessment using the fastQC tool available on the KBASE platform (Arkin et al., 2018).^4^ Assembly was performed with Unicycler v0.4.9 software (Wick et al., 2017), to produce 104 contigs with a mean coverage of 625×. Additionally, whole genome sequencing of *Virgibacillus flavescens* DJP025-4 was performed on PacBio Sequel IIe system by Macrogen Europe BV using the [3.0] PacBio Microbial library kit, producing 82,760 HiFi long-reads with an average length of 10,792 bp and quality Q30. These were assembled with Flye 2.8.3 (Kolmogorov et al., 2019) using default parameters for PacBio long-reads, resulting a single circular contig of 3,584,655 bp with GC content of 38.0% and coverage of about 50×. Illumina reads were also used to refine/confirm the correctness of PacBio assembly by using the Map to reference function inside Geneious Prime 2023.2.1.^5^ For genome-based phylogenetic reconstructions, 122 bacterial single-copy conservative marker genes were used as described previously (Parks et al., 2018; Rinke et al., 2021). Taxonomic classification and phylogenetic placement of the DJP025-4 genome within the GTDB reference tree were performed using the “classify_wf” workflow implemented in GTDB-Tk v. 2.5.2 (Chaumeil et al., 2022). The ANI screening step was skipped during the analysis. The DJP025-4 genome annotation and pathway prediction were conducted using several online tools: RASTtk Toolkit, and the NCBI Prokaryotic Genome Annotation Pipeline (PGAP v. 6.10) (Haft et al., 2024).

In addition, a differential methylome analysis was conducted to investigate the calcium-ion-specific stress response in *Vb. flavescens* DJP025-4 and to identify modified nucleotides. Two culture conditions were compared: growth in optimized Marine Broth supplemented with 1.2 M NaCl (MBSW70) and growth in MBSW70 medium supplemented with 1.0 M CaCl₂ (MBSW70 + 1 M). DNA methylation was analyzed using tools implemented in SMRT Link v.12.0.0.177059. PacBio reads in BAM format were mapped to the DJP025-4 assembled genome sequence using pbmm2 aligner, and the resulting alignments were sorted while preserving base-calling kinetic information. Methylated nucleotide positions were identified using ipdSummary based on kinetic variation in the aligned reads, together with estimates of local coverage and statistical significance. Methylation motifs were subsequently identified using motifMaker. The chromosomal distribution of methylated motifs was visualized using SeqWord Motif Mapper v.3.2.6,^6^ (Lefebvre et al., 2025), applying thresholds of ≥10 × coverage and QVAL ≥20 (*p* ≤ 0.01) for methylated sites. The program evaluates biases in the distribution of methylated and unmethylated motif occurrences across different functional genomic regions, including protein-coding sequences, non-coding regions, regions upstream of translation start codons, and genomic islands (GIs). Horizontally acquired genomic island were identified using SeqWord GISniffer v.3.1 (Bezuidt et al., 2009).^7^ The software was installed and executed on the high-performance computing cluster at the Centre for Bioinformatics and Computational Biology, University of Pretoria (Pretoria, South Africa).

### Determination of maximum salt concentrations suitable for DJP025-4 growth and survival

Bacterial growth in liquid nutrient medium MBSW70 with stepwise separate addition of NaCl (200, 215, 230, 245, and 265 g l^−1^) and CaCl_2_ (1.0, 1.25, 1.5, 1.75, and 2.0 M) was monitored by the increase in turbidity measured by the change in optical density at 600 nm during cultivation on a rotary shaker at 120 rpm for two to 3 weeks at 12 °C. In addition, the survival experiments using CaCl_2_ and sodium and magnesium perchlorates were monitored as colony-forming unit (CFU) counting. Initially, a 48-h culture of *Vb. flavescens* strain DJP025-4 grown in unmodified MBSW70 medium was collected by centrifugation and resuspended in 100 mL of 1.5 M NaCl solution (pH 7.4), achieving a final concentration of 1 × 10^8^ CFU/mL. The resulting suspension was then divided into 2 mL fractions, to which various salts with increasing concentrations were separately added: CaCl_2_ (0.75 M, 1.5 M, 2.75 M, and 3.0 M), NaClO_4_ and Mg(ClO_4_)_2_ (0.25 M, 0.5 M, 1.0 M, and 1.25 M for each salt). After a three-month incubation period at 12 °C in the presence of the aforementioned salts, CFU were counted after plating on Petri plates with MBSW70 agar using tenfold dilutions. The number of cells surviving exposure to 2.75 M and 3.0 M CaCl_2_ was counted after plating of undiluted samples (5–10 μL). The results were converted into percentages compared to the number of CFU obtained in control samples (1.5 M NaCl solution without the addition of any other salts) subjected to the same three-month exposure. All experiments were performed in triplicate.

### Scanning electron microscopy/energy-disperse X-ray spectroscopy

Cells grown in MBSW70 liquid nutrient medium supplemented with various CaCl_2_ concentrations were fixed with 2.0% (vol/vol) glutaraldehyde diluted in isotonic NaCl or CaCl_2_ solutions for 1 h. The fixative was removed with a single 5-min rinse in isotonic NaCl solution, followed by five successive 5-min washes with gradually decreasing salt concentrations, down to distilled water. The fixed material was placed onto poly-L-lysine-coated coverslips for 10 min to cross-link microbes with poly-L-lysine coating. Dehydrating was achieved using a series of increasing of ethanol in water mixtures [10, 30, 50, 70, 90% (v/v) ethanol] and pure ethanol for 10 min for each step. The samples were then subjected to critical-point drying with liquid CO_2_ (Quorum K850 Critical Point Dryer, Quorum Technologies Ltd., London, United Kingdom). The dried samples were covered with a gold film by sputter coating (Quorum Q150R S plus; Quorum Technologies Ltd., London, United Kingdom) before examination in a scanning electron microscope TESCAN Mira 3 (Tescan Brno s.r.o., Brno, Czech Republic) with an acceleration voltage of 5 kV. Additionally, scanning electron microscopy (SEM) mapping was conducted using a Nova Nano Scanning Electron Microscope, equipped with a Bruker energy-disperse X-ray spectroscopy (EDS) detector. The images were captured at a 5 nm resolution and a 100,000 × magnification. EDS analysis and SEM mapping were conducted following the procedures described in “ASTM standard method E1508–12a” (Das et al., 2014). The EDS analysis was conducted with a spot size of 5, an accelerating voltage of 20 kV, and an error rate of 4%.

### Electron cryotomography data collection and processing

To perform electron cryotomography (cryo-ET), DJP025-4 cells grown in MBSW70 liquid nutrient medium supplemented with various CaCl_2_ concentrations were fixed with 0.125% glutaraldehyde. Subsequently, cells were washed quickly in PBS. This step was done rapidly to prevent any lysis of cells due to changes in the osmolarity of the medium during washing. The cultures were subsequently mixed with 10 nm colloidal gold beads (Ted Pella, CA, United States) pre-coated with 1% BSA. About 4 μL of the sample mixture was added to a glow-discharged copper R2/2 Quantifoil holey carbon grid (Electron Microscopy Sciences, PA, United States). Grids were blotted using a Vitrobot (Mark IV, FEI Thermo Fisher Scientific) and plunge frozen in liquid ethane. Grids were imaged under cryogenic conditions, using a Transmission Electron Microscope (FEI Titan Krios G4, 300 KeV FEG; Thermo Fisher Scientific) equipped with a Gatan K3 Summit direct electron detector and an energy filter (GIF, Gatan) set to 20 eV. Tilt series were acquired in movie mode from −60° and +60° at 2° increments. The cumulative total dosage used was 100 e^−1^ Å^−2^, with a defocus of −8 μm and a pixel size of 3.4 Å. Tilt series were aligned using IMOD. The 2 K binned micrographs were then reconstructed using Tomo3D into 1 K SIRT tomograms (Agulleiro and Fernandez, 2011).

### mRNA extraction and analysis

All these procedures were described in detail in our previous publication (Reva et al., 2024, 2025). Briefly, total RNA was extracted from 5 mL of triplicate MBSW70 and MBSW70 + 1 M cultures at the late exponential phase using the MasterPureTM Complete DNA and RNA Purification Kit (Epicenter, VWR, Milan, Italy). Cells were harvested by centrifugation at 10,000 × g, 4 °C, for 20 min, and RNA extraction followed the manufacturer’s instructions. The RNA was resuspended in 50 μL of RNase-free water and treated with the TURBO DNA-free kit (Ambion, TX, United States) to remove residual DNA. RNA quality and concentration were measured using the Qubit 3.0 fluorometer (Thermo Fisher Scientific, Italy). DNA removal was confirmed by PCR amplification with S-D-Bact-0341-b-S-17 and S-D-Bact-0785-a-A-21 primers (Caporaso et al., 2012) and none PCR products were detected on a 1% agarose gel. RNA integrity was assessed using the Agilent 2,100 Bioanalyzer (Agilent Technologies). After rRNA depletion and DNase treatment, sequencing was performed at the Macrogen Europe Sequencing Centre (see text footnote 1) using the Illumina^®^ NextSeq platform with 2 × 100 bp paired-end libraries (NextSeq Reagent Kit v2.5). Using differential transcriptome analysis via Gene Ontology (GO) annotation of differentially expressed genes (DEGs), we were aimed to study Ca-specific stress response in *Vb. flavescens* DJP025-4 cells to elevated environmental level of this chaotrope. Two culture conditions were compared: growth in optimized Marine Broth medium supplemented with 1.2 M NaCl (MBSW70) and in MBSW70 medium supplemented with 1 M CaCl_2_ (MBSW70 + 1 M). This simplified experimental design allowed us to analyze in detail the effects of calcium stress on the physiological and functional responses of the model organism. Transcriptome analysis and quality checks were conducted with PRINSEQ-lite. Only differentially expressed genes with false discovery rate (FDR) ≤ 0.05 were considered.

### Quantification and statistical analysis

Transcriptional analysis was conducted using Bioconductor version 3.17 on R-3.4.4. RNA sequences were aligned against the reference genome of *Vb. flavescens* DJP025-4 (CP192260). The featureCounts in Bioconductor’s Rsubread v.2.0.1 package counted reads over coding sequences (CDS). DESeq2 v.1.26.0 and GenomicFeatures v.1.38.2 normalized counts by total reads and CDS lengths, analyzing gene expression, fold changes, and *p*-values across datasets. Gene regulation involving an absolute expression fold change of 2 or greater, with a *p*-value of 0.05 or less, was considered statistically significant. Fold change values of gene expression predicted by the program DESeq2 were used to calculate Pearson product–moment correlation of gene regulation using function stats.pearson of the library scipy v.1.11.2 in Python 3. The two-tailed *p*-value of Pearson correlation was calculated using function stats.t.cdf of the same library. Principal Component Analysis (PCA) algorithm, implemented in the PAleontological STatistics (PAST) 4.02 program,^8^ (Hammer et al., 2001) was used for clustering gene expression patterns. For the PCA analysis, reads per kilo base per million mapped reads (RPKM) values of gene expression were calculated as described previously (Reva et al., 2025). Before PCA clustering, row-scaling normalization of RPKM values for the same genes, determined under different growth conditions, was performed by dividing all values in each row by the highest RPKM value in that row. Row-scaling normalization is necessary to eliminate the dominance of highly expressed genes during PCA analysis. The program PAST was used to draw estimated 95% confidence ellipses for the distribution of gene expression patterns on the PCA plot. Statistical significance of differences between gene expression patterns was calculated using the Permutational Multivariate Analysis of Variance (PERMANOVA) algorithm (Anderson, 2014), implemented as the stats.distance function in the skbio v.0.6.2 library in Python 3 using the default settings of arguments (999 permutations).

## Results and discussion

### Location of the study area, sample collection, and initial analyses

During the XXXVIII PNRA Italian Antarctic Expedition (November 2022—January 2023), fieldwork, geochemical and microbiological characterization of the Don Juan Pond (DJP) basin located in Upper Wright Valley (McMurdo Dry Valleys, Antarctica) were conducted. Numerous ephemeral streams and shallow ponds were discovered in the western part of the DJP basin, near the central CaCl₂-rich brine (Figures 1A,B). Their formation likely results from the mixing of seasonal meltwater influx from nearby rocky glaciers with the DJP brine, which is fed by a CaCl₂-saturated underground aquifer. Interestingly, no minimal traces of similar hydrological features were observed the previous year, during the XXXVII Expedition (October 2021—January 2022).

**Figure 1:**
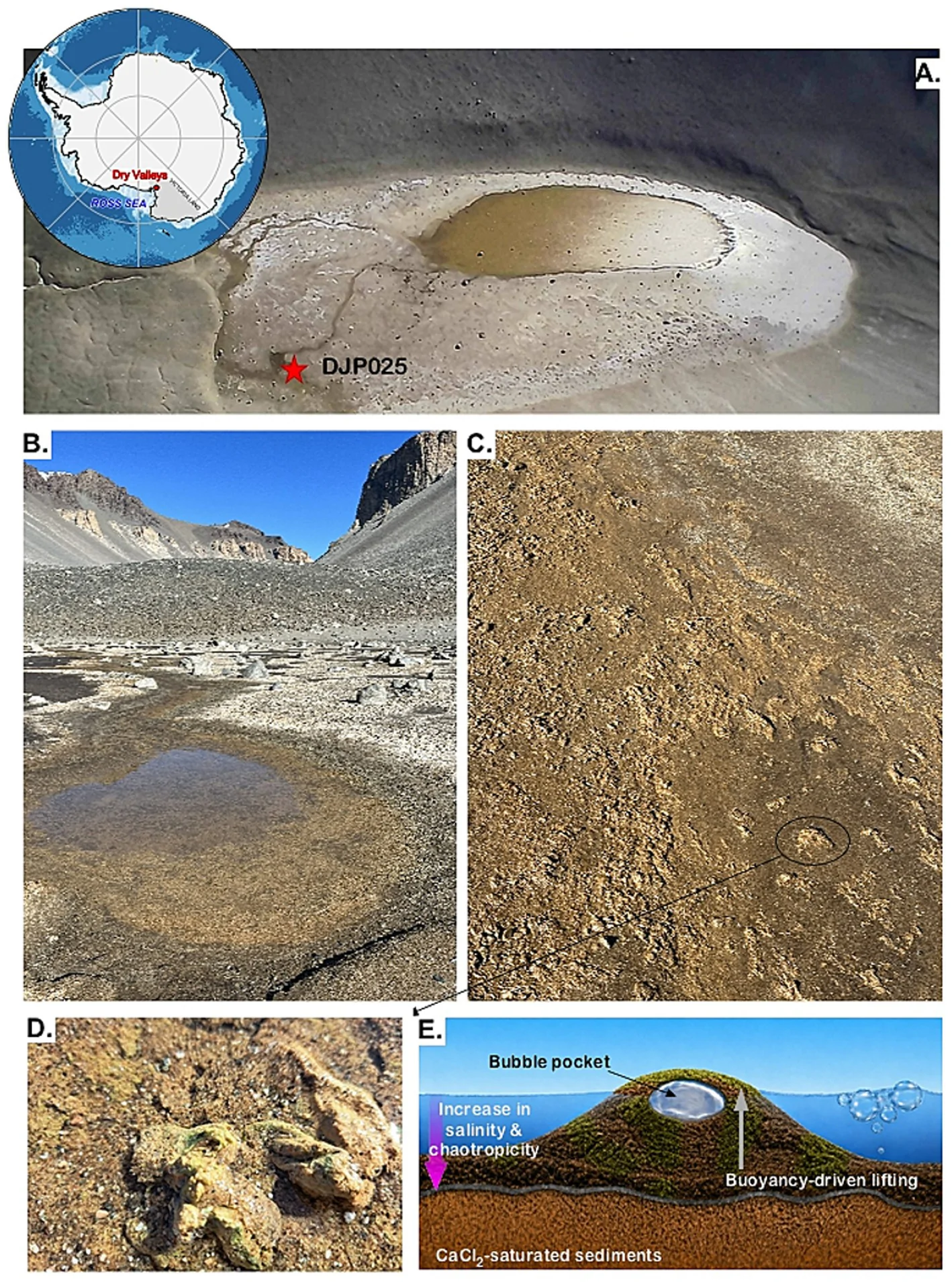
Aerial view of the study site, Don Juan Pond (DJP), in the Upper Wright Valley, McMurdo Dry Valleys, Antarctica, taken in November 2022 **(A)**. Numerous ephemeral streams and shallow ponds were found in the western part of the DJP basin, near the central CaCl_2_-rich brine **(A,B)**. Creeping microbial mats, observed during the visit on the western periphery of the DJP, resemble a crumpled, thin carpet, sometimes forming amorphous suspended structures due to the buoyant effect of bubbles trapped within the mat **(C,D)**. Simulation model of photosynthetic mats that trap oxygen-rich bubbles and become buoyant, lifting off the calcium chloride-rich bottom **(E)**. The locations of the Antarctic Dry Valleys and the sampling site DJP025 are shown in the inset and red star, respectively.

Initial measurements of surface salinity and temperature in one of the streams, designated DJP025, using a portable refractometer and the YSI multi-probe sensor revealed lower salinity (83.5 g l^−1^ total salinity) compared to the bottom part of the stream (300 g l^−1^ total salinity). Given the generally very shallow depth of DJP025 (≤5 cm), this significant salinity gradient is easily explained by the aforementioned phenomenon of mixing of two different water masses. Visual inspection of the DJP025 bottom revealed the presence of crust-like greenish-brown microbial pinnacle mats resembling a thin carpet covering the clay-sandy bottom (Figures 1C,D). Physical disruption of the pinnacles resulted in the release of bubbles, presumably generated by phototrophic activity. We reached this conclusion by visual analogy with underwater microbial mats growing in the nearby perennially ice-covered lakes Fryxell, Joyce, and Hoare in the McMurdo Dry Valleys of Antarctica (Juarez Rivera et al., 2025). These photosynthetic benthic mats trap oxygen-rich bubbles and become buoyant, tearing off the bottom as “liftoff mats” forming a sequence of morphological forms, starting with bubble-induced deformation of flat mats and progressing to tents, ridges, and pinnacles. We hypothesize that gas bubbles, even if short-lived, can exert a significant impact on the functioning of microbial mats at large scales in calcium-rich DJP brines (Figure 1E). Indeed, rising a portion of the microbial mat to the upper, less saline layers of the stream creates a much less hostile environment for it compared to the mat located at the bottom, where calcium concentrations exceed 2.5 M (Table 1). A portion of the collected microbial mat samples, delivered to the Mario Zucchelli Station, were immediately stored at 4 °C and transported to a laboratory in Italy for subsequent enrichment/cultivation attempts, in accordance with the basic hydrochemical conditions of the DJP025 stream (see the next chapter). Naturally, we collected a minimal amount of material, paying particular attention to the “Do No Significant Harm” (DNSH) principle, which applies to fieldwork activities aimed at preventing and controlling pollution, as well as protecting and restoring biodiversity/ecosystems.

**Table 1:** Major ion composition and other parameters of DJP sites and the most saline and anhydrous (a_w_ < 0.400) athalassohaline lakes on Earth.

	*Don Juan Pond* Ice	*Don Juan Pond* Site DJP025	*Don Juan Pond* Central area	*Discovery* Lake brine^a^	*Hephaestus* Lake brine^a^	*Kryos* Lake brine^a^
Major ions, mM						
Na^+^	4.39	29.57	47.83	84	93	125
K^+^	0.81	0.52	0.11	20	28	80
Mg^++^	4.02	5.81	3.29	5,150	4,720	4,379
Ca^++^	405	2,745	4,341	1	2	1
Cl^−^	811.3	5,515	8,716	10,150	9,120	9,043
SO_4_^−−^	1.18	1.82	1.21	110	203	320
NO_3_^−^	0.71	3.73	7.03	NA	NA	NA
Parameters						
Salinity, g l^−1^	45.32	307	485	510	480	471
Temperature, °C	−4.8	−1.2	−2.2	16.1	15.3	14.5
pH	6.1	5.9	~5.5	~4.5	~5.0	~5.4
Density	1.03	1.23	1.25	1.33	1.32	1.32
Water activity, a_w_	0.975	0.753	0.413	0.382	0.395	0.399
Chaotropicity^b^, kJ kg^−1^	37.3	252.8	399.7	259	227.2	195.6

^a^The composition of the Mg^++^-saturated Eastern Mediterranean deep-sea brine lakes Discovery, Hephaestus and Kryos is taken from La Cono et al. (2019). ^b^Chaotropicity values were calculated according to Cray et al. (2013, 2015), based on the abundance of dominant cations and anions and the charge-balanced mixture of ions was divided into corresponding equivalent salt combinations, assuming they essentially form chlorine and sulfate salts.

### Geochemistry of the DJP025 stream, ice, and the DJP central brine

The total dissolved solids content in the DJP025 stream, determined in a laboratory in Italy, was slightly higher than that measured *in situ* using a YSI multi-probe sensor (Table 1). For comparison purposes, the ice covering the stream at the time of our visit and the central DJP brine were also analyzed. The salinity values increased from the ice to the stream bottom, from 45.3 to 307 g l^−1^, corresponding to 0.41 and 2.75 M of Ca^2+^ and water activity (a_w_) of 0.980 and 0.753, respectively. However, the harsh environmental conditions in the DJP025 stream were significantly less hostile than the DJP central brine parameters, measured during sampling (salinity of 485 g l^−1^, Ca^2+^ 4.34 M, and a_w_ 0.425). Consistent with the available literature, the chemical composition of the DJP brine was predominantly represented by Ca^2+^ and Cl^−^, comprising 99.6% of the total cations and anions analyzed (Table 1). This dominance was maintained in both stream water (99.54%) and ice (99.3%), despite corresponding dilution or displacement of ions during freezing. Dilution processes had only minor effects on the relative Ca^2+^/K^+^, Ca^2+^/Mg^2+^, and Ca^2+^/SO_4_^2−^ molar ratios in the DJP025 stream compared to the DJP central brine (5.28 × 10^3^, 4.73 × 10^2^ and 1.51 × 10^3^ vs. 3.95 × 10^4^, 1.32 × 10^3^, and 3.59 × 10^3^, respectively), indicating a comparative enrichment of the glacial meltwater influx in these ions from the subsurface calcic brine feeding the DJP central region.

### Diversity assessment and profiling of DJP025 microbial mat community

General characteristics of the metabarcoding analysis obtained from the DJP025 microbial mat sample is summarized in Supplementary Table 1. A total of 27,009 16S rRNA gene sequences were obtained, representing 787 distinct amplicon sequence variants (ASVs), including 44 singletons. A total of seven bacterial phyla and three eukaryotic phyla (all unicellular phototrophs) were identified in the DJP025 microbial mats. No 16S rRNA gene sequences belonging to archaea were detected, even in analyses using archaea-specific primers (Bahram et al., 2019). Rarefaction curve with 99.9% coverage was obtained for the DJP025 ASV library, indicating adequate representation of microbial diversity. The large number of ASVs obtained is reflected in the alpha and beta diversity analysis, and all parameters, including the Shannon index, Chao1, and inverse Simpson index, showed high values (6.0, 800, and 294, respectively). However, a detailed examination of the obtained reads and their phylogenetic affiliations revealed that the high diversity is more likely due to 16S rRNA gene variations at the intrageneric or even intraspecific level. This fact explains the absolute dominance of representatives of only four genera, which accounted for approximately 40% of all analyzed reads: *Yoonia* (*Alphaproteobacteria*) -14.9%; *Gilissia* (*Bacteroidia*)—11.1%; *Luteolibacter* (*Verrucomicrobiia*)—9.1% and *Virgibacillus* (*Bacilli*)—4.7%. Among the seven bacterial phyla detected, *Pseudomonadota* (formerly *Proteobacteria*), *Bacteroidota*, and *Bacillota* were among the most abundant (>10%) bacterial phyla, accounting for more than 85% of all taxonomically analyzed 16S rRNA gene sequences (59.3, 15.6, and 10.3%, respectively) (Supplementary Table 1). Excluding bacterial phyla, the small number of 16S rRNA gene sequence reads (0.4%) detected in the DJP025 mat were more than 99% identical to chloroplast sequences obtained from various Antarctic glacier and polar sea ice samples and were distantly related to chloroplasts of diatoms, foraminifera, and green microalgae of the class *Ulvophyceae*.

### Isolation of Ca^2+^-resistant bacterial strains from the DJP025 microbial mat

Following arrival of the DJP025 microbial mat sample in laboratory in Italy (June 2023), we conducted enrichment experiments in a culture medium prepared according to DJP025’s main hydrochemical parameters (see Experimental procedures). All primary enrichments obtained from collected microbial mat material were assessed as positive (measured as increase in optical density at OD_600 nm_) within 1 month of cultivation at 10 °C. All were further converted to a microbial mat-free stage after three successive subcultures at a ratio of 1:100 (vol/vol) on the same culture medium. The enrichment, grown at a CaCl_2_ concentration of 1.0 M of and a total salinity of 150 g l^−1^, was subjected to a serial dilution-to-extinction (up to 10^−7^) procedure to obtain individual colonies on solid medium, and a total of four different Ca^2+^-tolerant microbial strains were isolated. The 16S rRNA gene sequences were aligned to the SILVA reference sequence using the built-in ARB aligner and manually checked for correct placement within conserved regions. Based on this analysis, all of these bacterial isolates were assigned taxonomic affiliations with 97–99% 16S rRNA gene sequence identity to previously described species: *Sutcliffiella horikoshii* DJP025-1, *Vreelandella* (formerly *Halomonas*) *glaciei* DJP025-2, *Virgibacillus arcticus* DJP025-3, and *Virgibacillus flavescens* DJP025-4 (Figure 2A). The latter isolate was 99.87% identical to the type strain *Vb. flavescens* SA-1^T^ (NR_148785), isolated from subsurface sediments of the Yellow Sea (Zhang et al., 2016).

**Figure 2:**
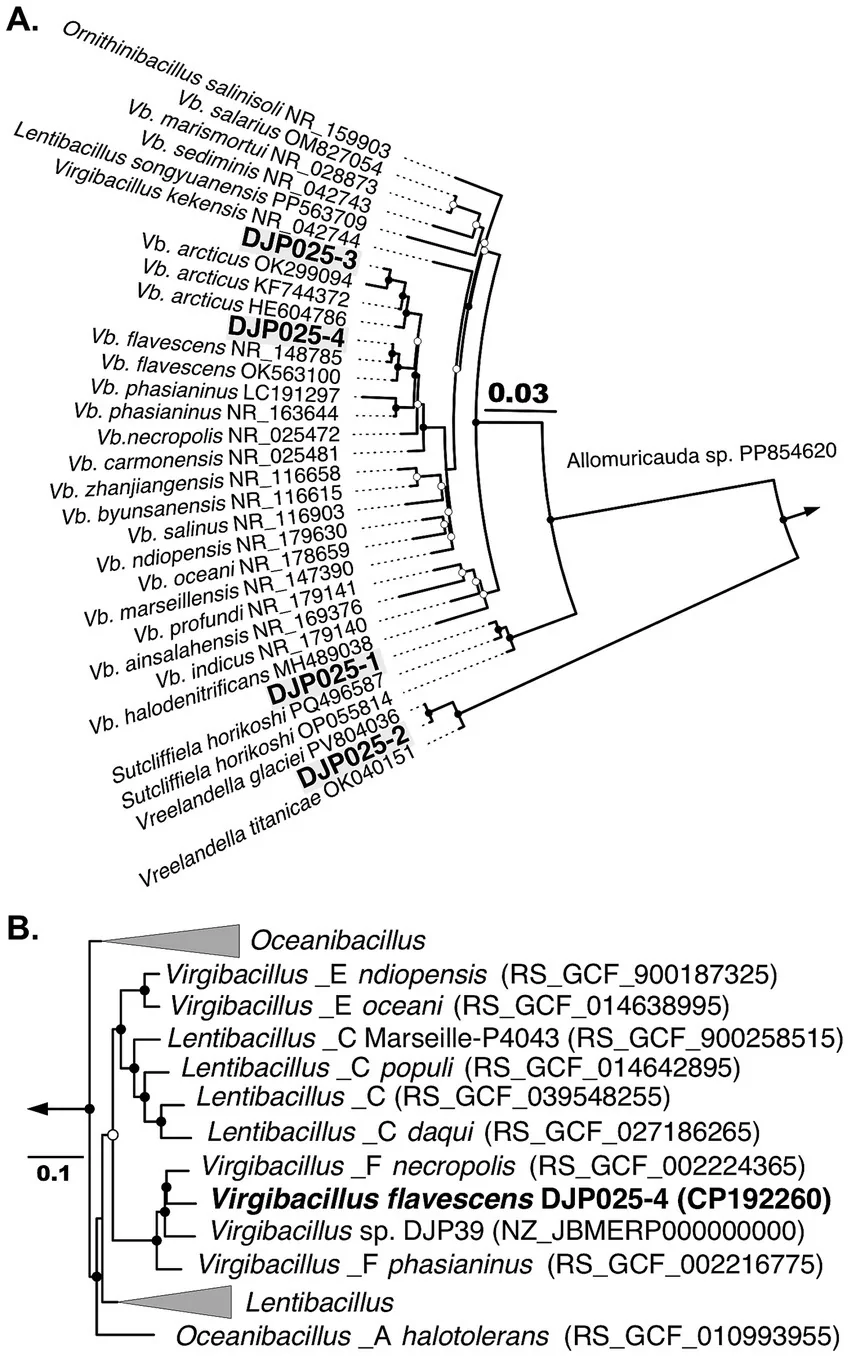
**(A)** Rooted phylogenetic tree based on 16S rRNA gene sequence showing the diversity of bacteria isolated from an enrichment of the DJP025 microbial mat supplemented with Ca^2+^ 1.0 M. The tree was rooted using the 16S rRNA gene sequence of the flavobacterium *Allomuricauda* sp. PP854620. **(B)** Maximum likelihood phylogenetic tree based on 122 conserved protein markers showing the position of *Vb. flavescens* DJP025-4 among closely related sequenced bacilli. Taxonomic designations correspond to the Genome Taxonomy Data Base (GTDB). Trees were constructed using IQ-TREE 2 with the approximate likelihood ratio test for branches. Branch lengths correspond to the number of substitutions per site with corrections associated with the models. Black and open circles at nodes in both trees indicate that the percentage of corresponding support values was >70 and >50, respectively.

According to hydrochemical analysis of the DJP stream surrounding the microbial mat from which the strains were isolated, the Ca^2+^ concentration there was in the range of 1.05–2.75 M (see above). This was taken into account when determining the range of Ca^2+^ concentrations in the culture medium that the strains could tolerate and grow in. All isolates were rated positive at CaCl_2_ concentrations up to 1.25 M (Figure 3A). *Vreelandella glaciei* DJP025-2 refused to grow at higher concentrations, while *Sutcliffiella horikoshi* DJP025-1 did not tolerate concentrations above 1.5 M. Both *Virgibacillus* isolates were rated as extremely calcium-tolerant, with only *Vb. flavescens* DJP025-4 proving the most interesting, demonstrating the ability to grow, although very slight, at a Ca^2+^ concentration of 2.0 M and total salinity of 180 g l^−1^ (Figure 3A), a level completely unattainable for all bacteria known before its discovery.

**Figure 3:**
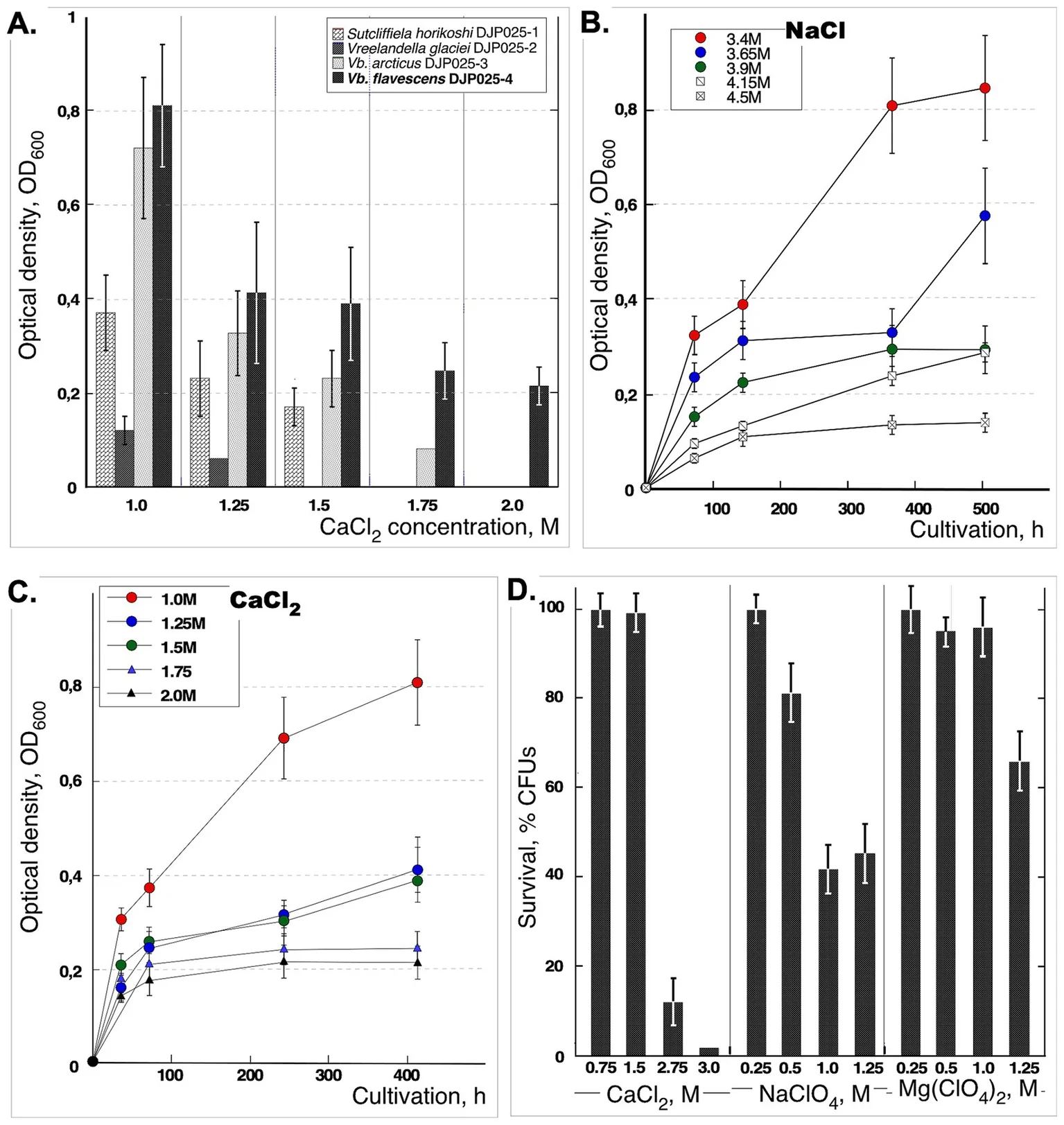
The maximum tolerable concentrations of CaCl_2_ suitable for the growth of selected DJP025 isolates are expressed as the maximum optical density (OD_600_) values achieved during 500 h cultivation at 12 °C **(A)**. Growth curves of *Vb. flavescens* DJP025-4 in MBSW70 liquid culture media supplemented with different concentrations of NaCl **(B)** and CaCl_2_ **(C)**. The survival potential of *Vb. flavescens* DJP025-4 at different concentrations of chaotropic agents such as CaCl_2_ and perchlorate salts NaClO_4_ and Mg(ClO_4_)_2_, expressed as a percentage of CFU formed by the end of the 3-month experiment and compared with the number of colony-forming cells exposed for the same time in 1.5 M of NaCl **(D)** (see section Experimental procedures). Error bars are given for three biological replicates.

### Growth and survival of *Vb. flavescens* DJP025-4 in kosmo- and chaotropic brines

Consistent with the observed extreme tolerance toward elevated calcium concentrations, we further determined how the growth and survival of strain DJP025-4 are affected by various kosmotropic (NaCl) and chaotropic (CaCl_2_ and perchlorates) brines. Increasing the NaCl concentrations to 3.40 M resulted in a slight decrease in the specific growth rate compared to optimal growth conditions in MBSW70 culture medium (corresponding to 1.5 M NaCl) at 12 °C (0.038 versus 0.034 h^−1^). However, with an increase in NaCl concentration to 230 g l^−1^ (corresponding to 3.90 M NaCl), the mean specific growth rate of DJP025-04 decreased to 0.01 h^−1^ and at a NaCl concentration of 265 g l^−1^ (4.50 M), growth virtually ceased (0.004 h^−1^) (Figure 3B). Sufficient growth of strain DJP025-4 in MBSW70 supplemented with CaCl_2_ was observed up to a concentration of 1.50 M (corresponding to a_w_ ≈ 0.86 and chaotropicity of 124.5 kJ kg^−1^), where the hourly continuous growth rate was 0.0042–0.0045 h^−1^. Higher concentrations of calcium in the range 1.75–2.00 M (corresponding to a_w_ ≈ 0.84–0.85 and chaotropicity of 148–170 kJ kg^−1^), caused a slight increase in optical density at 600 nm to 0.243 ± 0.037 during the only first 4 days of cultivation, which remained virtually unchanged until the end of experiment (18 days) (Figure 3C).

In light of this observation, namely the presence of non-growing but still viable cells at such high salt concentrations, we considered it necessary to investigate the survival aspect of DJP025-4 cells in chaotropic brines either Ca^2+^ or perchlorates. This is particularly interesting given that bacilli similar to DJP025-4 were recently isolated from sediments (14–16 cm depth) sampled by push coring in the central part of the DJP, where they apparently existed in a highly resistant spore form (Shaffer et al., 2026). Survival potential was monitored by counting colony-forming units (CFUs) after 3 months of exposure of DJP025-4 cells (initial concentration 1 × 10^7^ CFU mL^−1^) in mineral media (1.5 M NaCl, pH 7.2) containing either CaCl_2_ or perchlorates. After colony counting, the result was expressed as a percentage of the number of CFU formed at the end of experiment and compared with the number of colony-forming cells exposed to 1.5 M NaCl alone for the same time (see Experimental procedures). Overall, remarkable resilience was demonstrated, surpassing results previously obtained for other prokaryotes. *Vb. flavescens* DJP025-4 cells (or, more likely, spores) retained viability and culturability (albeit at a level of 12 ± 5% relative to the calcium-free control) after 3 months of exposure to 2.75 M Ca^2+^ ions (Figure 3D). Notably, surviving individuals were observed even after 3 months of exposure to Ca^2+^ at a 3.0 M solution, corresponding to a water activity (a_w_) of approximately 0.653 accordingly to the Pitzer equations and molal osmotic coefficient *φ*(CaCl_2_) (Teng and Lenzi, 1974; Staples and Nuttall, 1977; Rard and Clegg, 1997; Bui et al., 2003) and a total chaotropicity of 262.8 kJ·kg^−1^, the thermodynamic equivalent similar to that of the Lake Discovery brine (Table 1; Hallsworth et al., 2007; Cray et al., 2013, 2015). Thus, survival (albeit, most likely in the form of highly resistant spores) occurred under conditions well below the current window for chaotropicity equivalent (124.2 kJ·kg^−1^ at a_w_ = 0.79), characteristic of the 2.3 M of Mg^2+^interface layers in Discovery, Hephaestus and Kryos lakes (Hallsworth et al., 2007; Yakimov et al., 2015, La Cono et al., 2019). The survival and growth ability of DJP025-4 under NaClO_4_ and Mg(ClO_4_)_2_ were also assessed, and again the selected strain exhibited notable resistance to whole range of perchlorate concentrations applied (Figure 3D).

### DJP025-4-induced calcite precipitation

Such a remarkable resistance of *Vb. flavescens* DJP025-4 toward elevated concentrations of calcium were further studied in detail. Among a number of metabolic adaptations aimed at increasing calcium tolerance, its extracellular precipitation is the most widespread in the microbial world (Banks et al., 2010; Dhami et al., 2013). This process, called (bio)calcification or microbially induced calcium precipitation (MICP), plays a key role in the survival and development of microorganisms in extreme, hypersaline, and chaotropic environments. The genus *Virgibacillus* is widespread in hypersaline or Mg/Ca-enriched ecosystems, such as Dohat Faishakh and Khor Al-Adaid sabkha environments (Qatar), Covadura and C3 gypsum caves of Sorbas (Spain), caves of the Buda thermal karst system (Hungary) where these bacteria are able to form carbonate minerals even at high salt concentrations and low water activity (Borsodi et al., 2012; Silva-Castro et al., 2015; Disi et al., 2019; Abdelsamad et al., 2022; Yan et al., 2022; Martin-Pozas et al., 2025; Naja et al., 2025). Based on these results, we investigated the calcification capacity of isolate DJP025-4 using energy-dispersive spectroscopy (EDS) to analyze the elemental composition and distribution around microcolonies formed in calcium-rich cultures. Furthermore, DJP025-4 cells grown under these conditions were analyzed using scanning electron microscopy (SEM) and cryo-electron tomography (cryo-ET). The EDS analysis revealed that DJP025-4 grown at 1.25–1.75 M CaCl_2_ formed microcrystalline shells surrounding the microcolonies (Figure 4). Elemental distribution maps of these mineral coatings clearly demonstrated that calcium, but not sodium, is deposited on the cells. The by-product precipitation of NaCl from the medium during sample preparation clearly demonstrated that sodium precipitates outside the cells, and calcium and chlorine are virtually unbound. Оverall, the EDS X-ray spectra and, in particular, the carbon and oxygen distribution maps (Figure 4B) indicate that the calcium there is bound to carbonate rather than chlorine.

**Figure 4:**
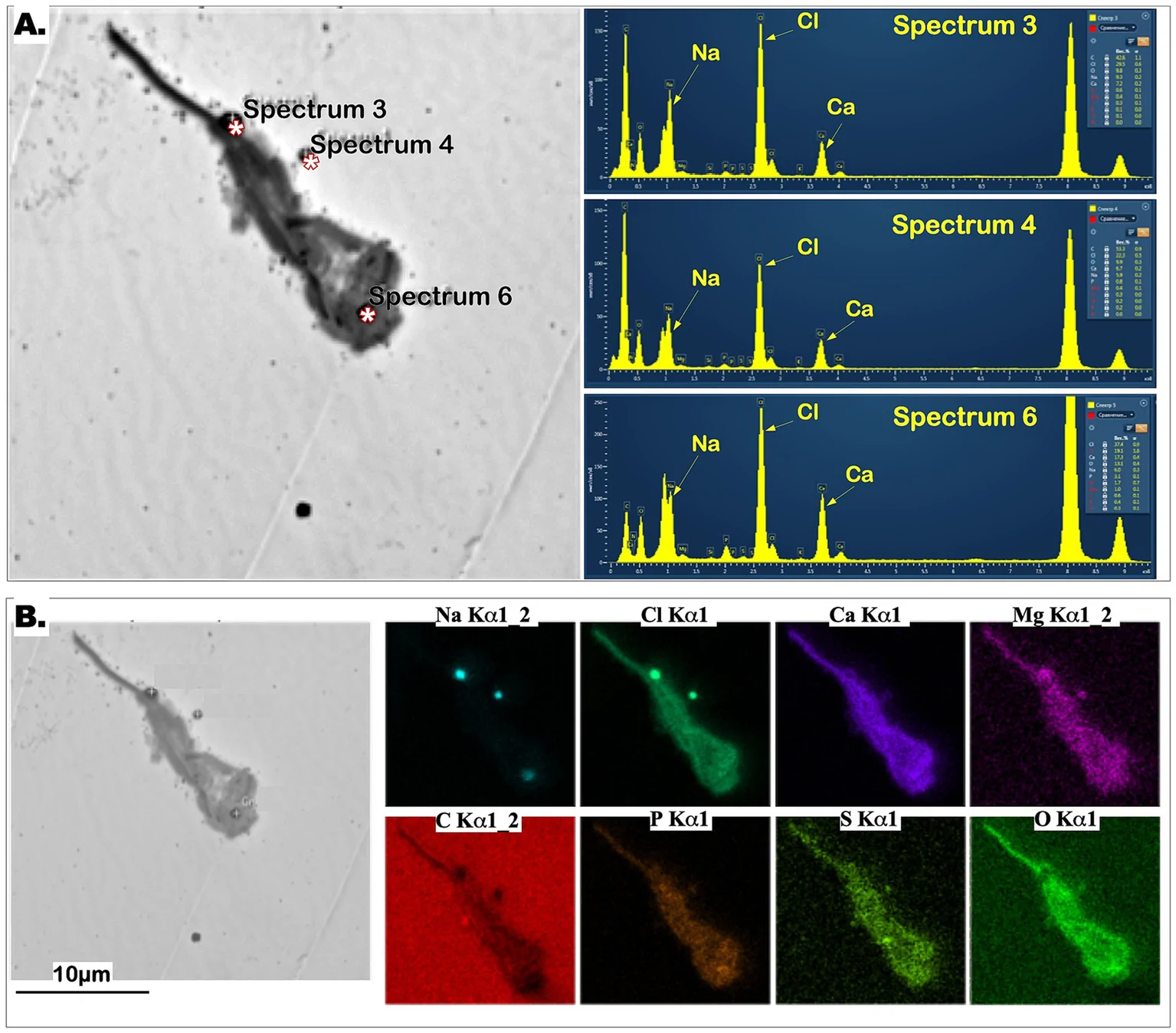
Energy-dispersive spectroscopy (EDS) of a *Vb. flavescens* DJP025-4 microcolony grown in MBSW70 medium supplemented with 1.50 M CaCl_2_. **(A)** Image of an EDS map (left) obtained using EDS with three selected spectra: Spectrum 3 and 4 (mineral precipitates) and Spectrum 6 (DJP025-4 microcolony). The energy spectra of these EDS maps and the detected elements (right). **(B)** The same EDS-EDS image (left) showing the detected elements (right): Top row—element map of sodium, chlorine, calcium, and magnesium; bottom row—element map of carbon, phosphorus, sulfur, and oxygen.

As shown in SEM images, compared to the morphology of control cells grown in the absence of calcium, DJP025-4 cells exhibited distinct bacterial encapsulation in response to the addition of Ca^2+^, continuing to grow being coated with precipitated layers of carbonate structures (Figure 5). SEM micrographs further demonstrate the formation of calcite encrustation over filamentous structural matrices of extracellular polymeric substance (EPS), which may form a kind of mineral bridge or complex network between adjacent cells (Figures 5C,D). When cultured in higher CaCl_2_ concentrations (1.75 M), calcite forms more intensely and, in addition to deposition on the cell wall, also accumulates at the cell ends as large of framboidal or spherical aggregations typical of biovaterite, a form of crystallized carbonate stabilized by EPS or glycoproteins in living organisms (Caicedo-Pineda et al., 2018; Yan et al., 2022).

**Figure 5:**
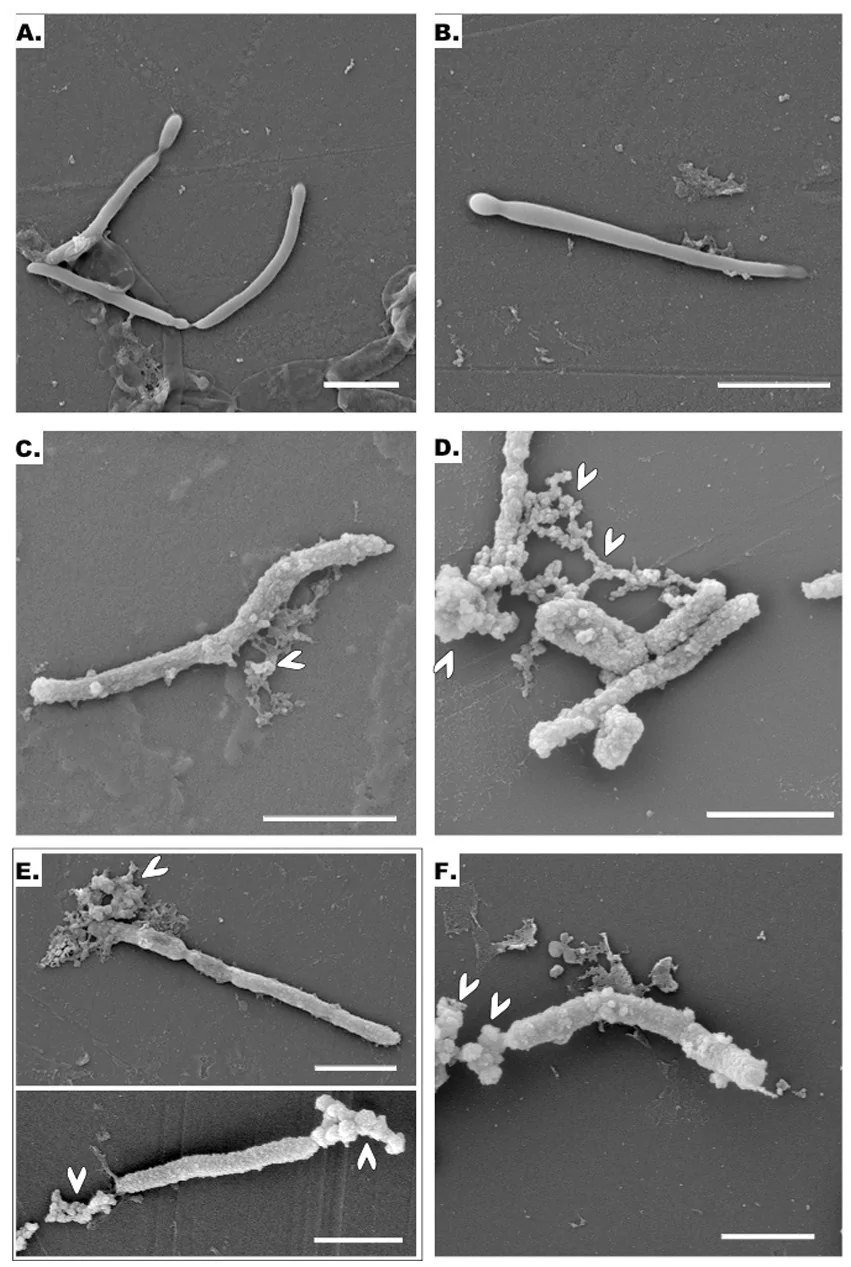
Scanning electron microscopy (SEM) images of calcite precipitation induced by *Vb. flavescens* DJP025-4. Cells were grown in calcium-free MBSW70 **(A,B)**, MBSW70 supplemented with 1.25 M **(C,D)** and 1.75 M of CaCl_2_ **(E,F)**, respectively. The location of framboidal and spherical clusters of biovaterite-like mineral away from the cells is indicated by white arrows. Scale bars represent 2 μm.

Cellular ultrastructural changes of *Vb. flavescens* DJP025-4 in response to calcium addition was obtained using cryo-ET, revealing interesting mechanisms of adaptation. Firstly, compared to the morphology of control cells grown in the absence of calcium, cells in the modified culture medium exhibit a clear visible thickening of the cell wall from 15 to 40 nm (Figures 6A,B), likely due to an increase in both the peptidоglycan layer and the EPS matrix. This apparently results from protection against calcium toxicity by hindering its penetration into the cells. Specifically, thickening of the cell wall at the apices (up to 55 nm) was observed in majority of analyzed cells exposed to CaCl_2_ (Figure 6A). Polar thickening of the cell wall is known to help to withstand high osmolarity outside the cells and the associated mechanical stress during directed cell elongation and division (Han et al., 2023). This is particularly important for strain DJP025-4 due to the presence of a hard calcite shield, which forces the cells to assume a thin, elongated shape.

**Figure 6:**
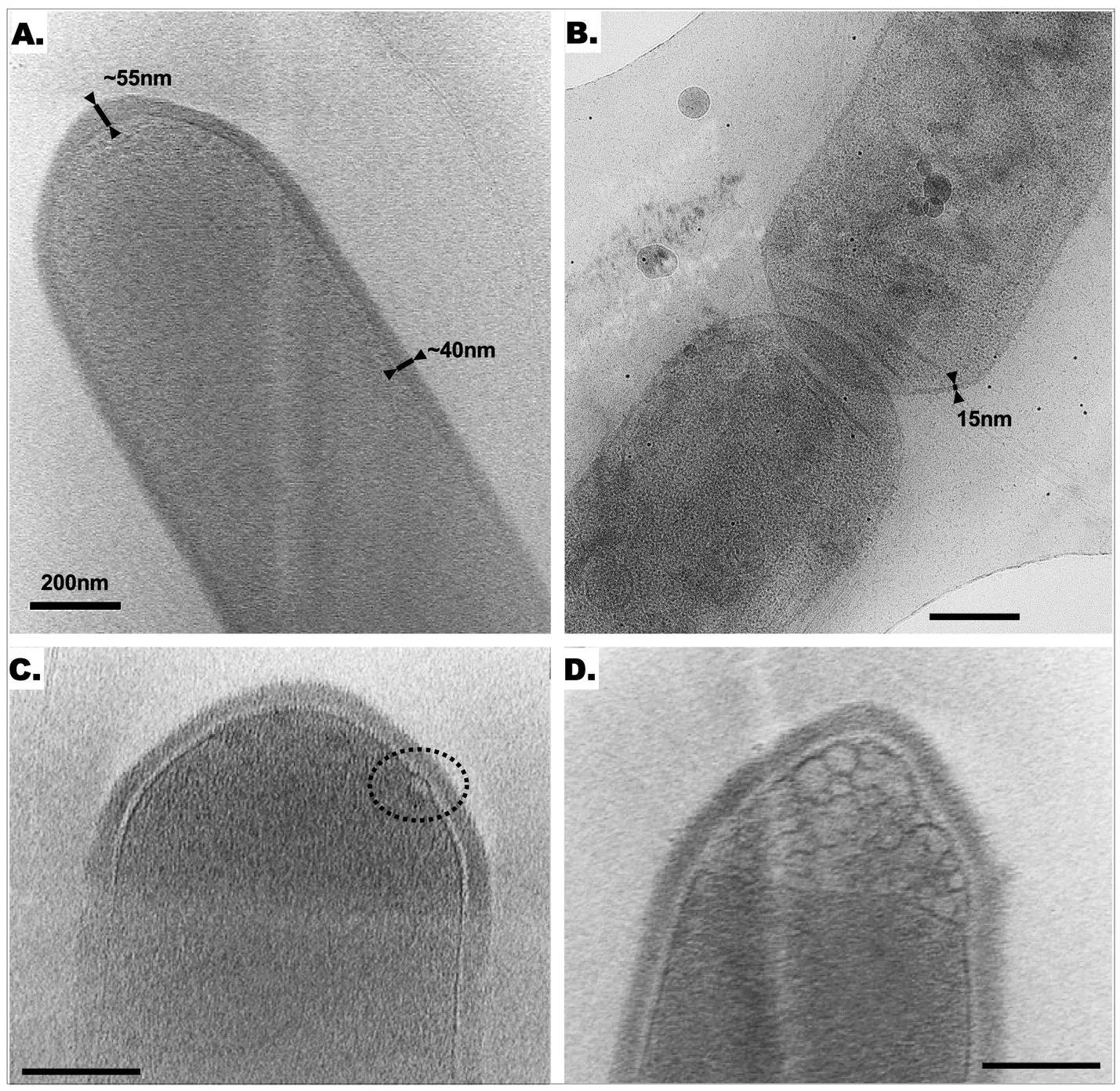
Cryo-electron tomography (Cryo-ET) images of *Vb. flavescens* DJP025-4 cells grown in MBSW70 medium supplemented with CaCl_2_ (0.75 M) **(A)** and without calcium **(B)**, respectively. Compared to control cell morphology, cells in the modified culture medium exhibit a clearly visible multiple-fold thickening of the cell wall, apparently resulting from adaptation and resistance to calcium toxicity **(A–D)**. The cell wall thickening at the apices was observed in most analyzed cells exposed to CaCl_2_. The initial formation of tentative polyhydroxyalkanoate (PHA) granules (highlighted by the dotted circle in **C**) and their subsequent accumulation at the cell poles **(D)** are clearly visible. Scale bars represent 200 nm.

Secondly, polyhydroxyalkanoate (PHA)-like granules were frequently detected in cells grown with the addition of calcium, whereas these granules were virtually absent in control cells. It is noteworthy that the initial formation of polyhydroxyalkanoate (PHA) granules and their subsequent accumulation occurred exclusively at the cell poles (Figures 6C,D). It is well known that the accumulation of polyhydroxyalkanoate (PHA) granules at the poles of microbial cells is a genetically and structurally regulated process that optimizes bacterial division and carbon reserve allocation, ensuring population survival (Peters et al., 2007). Indeed, during binary cell division, granules anchored at opposite poles are guaranteed to separate into two daughter cells, and each new bacterium immediately gains access to a carbon and energy source under stressful conditions (Jendrossek, 2009).

### Genome assembly and identification of horizontally acquired genomic islands

The genome of *Vb. flavescens* DJP025-4 was sequenced, assembled, annotated by the NCBI Prokaryotic Genome Annotation Pipeline (PGAP v. 6.10) (Haft et al., 2024), and deposited in NCBI data base under accession number CP192260. The genome consisted of a single circular chromosome of 3,584,655 bp in length, containing 3,650 genes, 3,564 coding sequences (CDS), 63 tRNAs, 4 ncRNAs (non-coding RNA), 1 tmRNA (transfer-messenger RNA or 10Sa RNA) and 6 complete rRNA operons (16S-23S-5S) (Table 2).

**Table 2:** General information for *Virgibacillus flavescens* DJP025-4 genome.

Attribute	Value
Genome composition	1 circular chromosome
Genome size	3,584,655 bp
GC content	38.0%
DNA coding region (%)	3,059,527 bp (85.3%)
Total genes	3,650
tRNA genes	63
rRNA genes (5S-16S-23S)	18
ncRNA genes	4
tmRNA genes	1
CRISPR repeat region	–
Protein-coding genes	3,564
Average gene length	848.9 bp
Max gene length	4,728 bp

Locations of putative horizontally acquired DNA regions were predicted using SeqWord GISniffer, which compares local tetranucleotide usage patterns with the genome-wide pattern (Bezuidt et al., 2009). In total, 23 putative genomic islands (GIs) were identified. The GIs were distributed unevenly across the chromosome, with the highest concentration occurring on the lagging replichore in the fourth chromosomal quartile, when chromosome coordinates were considered from the origin of replication through the replication terminus and back to the origin (Figure 7). The lagging replichores of bacterial chromosomes have been reported to be enriched in genomic islands and accessory genes and to exhibit greater evolutionary plasticity than origin-proximal regions of the leading replichores (Lato and Golding, 2021).

**Figure 7:**
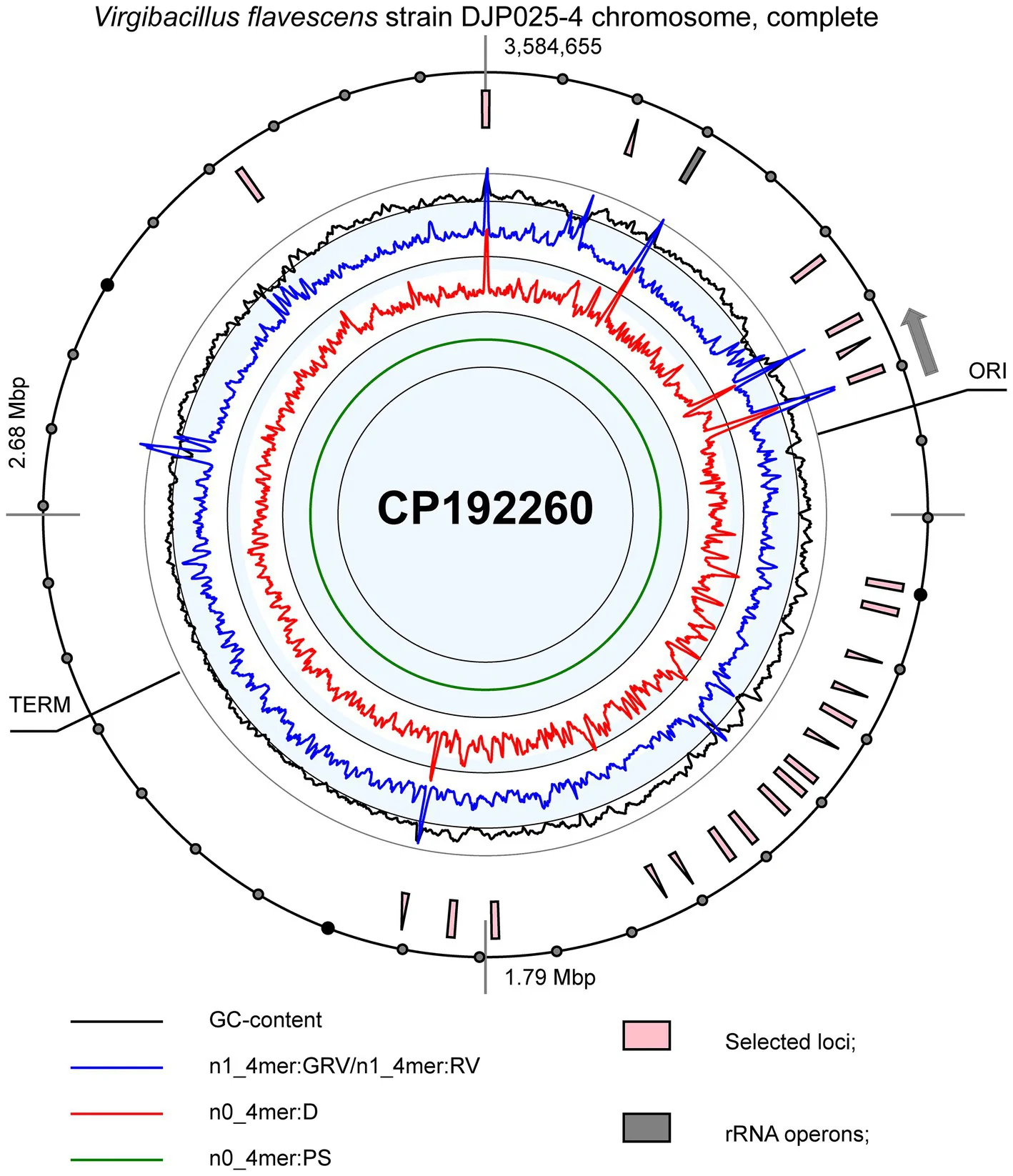
Distribution of chromosomal regions with atypical tetranucleotide usage patterns predicted by SeqWord GISniffer as putative horizontally acquired genomic islands (GIs). The locations of predicted GIs on the circular chromosome of *Vb. flavescens* DJP025-4 are indicated by pink blocks. Local variations in GC content and tetranucleotide usage parameters used for GI prediction are shown as colored line plots. Location of the replication origin (ORI) and replication terminus (TERM) are indicated. The leading replichore direction is indicated by a black arrow.

The analysis of important metabolic genes located within the predicted GIs is presented in Table 3. No plasmid integrases, transposases, or phage-related genes were identified within these regions or at their boundaries, suggesting that natural transformation of extracellular DNA may be a major mechanism of horizontal gene transfer in this environment. This hypothesis is further supported by the presence of key central metabolic genes within these GIs, which are not typically associated with phage- or plasmid-borne mobile genetic cassettes. Natural genetic transformation is an important adaptation mechanism employed by extremophilic bacterial communities, facilitating rapid adaptation to changing and harsh environmental conditions (Averhoff et al., 2021). In *Thermus scotoductus*, it was demonstrated that the uptake of naked environmental DNA originating from microorganisms with different degrees of relatedness, followed by the incorporation of DNA segments encompassing entire functional operons, contributes substantially to the adaptation and evolution of extremophilic bacteria (Gounder et al., 2011). Numerous clusters of central metabolism genes in the genome of *Vb. flavescens* DJP025-4 suggests exploiting similar adaptation strategies by the bacterial consortium of Don Juan Pond.

**Table 3:** Genomic islands identified in the chromosome of *Vb. flavescens* DJP025-4 and their functional annotation.

Island	Coordinates	Important genes/pathways	Likely significance
GI 1	209,444–221,553	*purD, purH, purN, purM, purF, purL, purQ, purS, purC*	A nearly continuous *de novo* purine/IMP biosynthesis cluster.
GI 2	518,755–530,351	*tenA, thiW, thiE, thiM, thiD*	Thiamine (vitamin B1) metabolism/biosynthesis and transport genes.
GI 3	613,901–626,099	*rocF, mvk, mvaD*, phosphomevalonate kinase, HMG-CoA reductase	Mevalonate/isoprenoid pathway genes and also arginine catabolism via RocF arginase and K^+^ uptake via TrkH.
GI 6	989,672–1,001,408	*gltB*, glutamate synthase β subunit, *wrbA*	Glutamate biosynthesis/nitrogen metabolism, with a NAD(P)H:quinone oxidoreductase.
GI 7	1,023,925–1,035,776	phenylacetate-CoA ligase, *paaX*, *paaA-D*, enoyl-CoA hydratases, 3-hydroxyacyl-CoA dehydrogenase, thiolase	Phenylacetate degradation genes.
GI 8	1,099,496–1,108,768	*hutH, hutU, hutI, hutG*	Nearly complete histidine utilization (*hut*) pathway, converting histidine through urocanate toward glutamate.
GI 9	1,149,616–1,158,203	*speB*, acetyl-CoA C-acetyltransferase, 3-hydroxybutyryl-CoA dehydrogenase	Agmatine/polyamine metabolism plus acetyl-CoA/3-hydroxybutyryl-CoA metabolism.
GI 10	1,185,466–1,196,513	*rpiB, glyA, upp, wecB, atpB*	Diverse central metabolism: pentose phosphate metabolism, one-carbon/serine metabolism, pyrimidine salvage, cell-envelope sugar metabolism and ATP synthase.
GI 12	1,276,672–1,293,387	*nikB*, multiple ABC transporter components, *ggt*, chromate transporters	Strong metal/nutrient acquisition and detoxification genes: nickel uptake, chromate transport and glutathione metabolism.
GI 14	1,335,154–1,350,214	aminotransferases, glycosyltransferases, acyltransferases, sugar transferase	Large cell-surface polysaccharide/glycan biosynthesis cluster potentially important for cell-envelope composition.
GI 15	1,394,000–1,404,703	glycerate kinase, *putP*, *gntP* permease	Glycerate/carbon metabolism plus proline and other metabolite transport.
GI 17	1,500,000–1,508,748	carbohydrate ABC transporter + several Gfo/Idh/MocA and sugar isomerase/epimerase proteins	A coherent carbohydrate uptake and utilization island.
GI 18	1,538,798–1,546,951	*hpaE, hpaB, hpaD*, flavin reductase, *pdhA*	Genes involved in 4-hydroxyphenylacetate/aromatic compound degradation, coupled to oxidative and keto-acid metabolism.
GI 19	1,773,063–1,784,099	Zn/Mn transporter, heavy-metal P-type ATPase, *copZ*, proline dehydrogenase, acyl-CoA dehydrogenase	Metal homeostasis/detoxification combined with amino-acid and fatty-acid metabolism.
GI 20	1,833,295–1,844,279	*allB*, *ubiX*, *ubiD*, adenine deaminase, BMC proteins	Genes involved in allantoin/purine catabolism and a probable bacterial microcompartment (BMC) system.

### Phylogeny and functional genome analysis of *Vb. flavescens* DJP025-4

Using the widely used approach proposed by GTDB, a maximum-likelihood phylogenomic tree, based on a comparison of 122 conserved single-copy protein markers, was constructed to determine the taxonomic position of DJP025-4. The resulting placement confirmed the assignment of strain DJP025-4 to the genus *Virgibacillus* in subgroup F, which includes the species *Vb. necropolis* (RS_GCF_002224365) and *Vb. phasianinus* (RS_GCF_002224365). It is worth noting that we also performed the phylogenomic placement of the *Virgibacillus* sp. DJP39, whose unassembled genome (212 contigs, estimated genome size 4,217,496 bp) is available in GenBank under the accession number (NZ_JBMERP000000000, Shaffer et al., 2026). Using the same GTDB approach, we found that this DJP isolate also belongs to subgroup F, forming a tight cluster with *Vb. necropolis* and our strain DJP025-4 (Figure 2B).

Based on the observed phenotypic features, namely the pronounced tolerance of strain DJP025-4 to elevated Ca^2+^ concentrations, the genomic determinants of ionic homeostasis, osmotolerance and extracellular deposition of calcium carbonates were clarified. To survive in a CaCl_2_-rich DJP environment, strain DJP025-4 employs a compatible solute accumulation strategy, known as the “salt-out” strategy. The DJP025-4 genome possesses the full genetic potential for synthesizing glycine betaine from choline via the conservative *bet*AB-*cud*CT operon (VBD025_04585–600), whereas we failed to identify any genes involved in *de novo* choline synthesis. This indicates a probable dependence of the strain (auxotrophy) on an external source of choline, which it can assimilate with high efficiency due to the presence in its genome of at least 18 ABC-type transport systems belonging to three specialized types of osmoregulatory transporters (Opu, Pro and BCCT systems). The DJP025-4 genome encodes an extensive network of Na^+^/H^+^ (12 genes), K^+^/H^+^ (VBD025_08860) antiporters, specialized Ca^2+^/H^+^ antiporters of the ChaA family (VBD025_04385 and VBD025_17755), and calcium-translocating P-type ATPase of SERCA-type (VBD025_12775) that ensure the active extrusion of calcium ions. This is of particular importance, since regulation of intracellular concentration of Ca^2+^ is a critical task; the level of this cation in the cytoplasm typically must be maintained in the nanomolar range (<10^−7^ М) to prevent the precipitation of macroergic phosphates (DNA, ATP) (Chandra et al., 2021).

Particular attention was paid to studying the mechanism of highly efficient extracellular CaCO_3_ precipitation induced by strain DJP025-4. The main known microbial metabolic pathways involved in this biocalcification process include urea hydrolysis, amino acids ammonification, denitrification, photosynthesis, methane oxidation, sulfate reduction; all in combination with oxidation of organic compounds (Vincent et al., 2021 for additional references). These metabolic pathways share common factors: CO_2_ production, the presence of Ca^2+^ cations, and local alkalinization, which promotes (bi)carbonate formation and CaCO_3_ precipitation. Among these metabolic pathways, urea hydrolysis is the most known and efficient biocalcification process in environments where urea is present (Tepe et al., 2019; Vincent et al., 2021; Naja et al., 2025). Although this pathway is undesirable in marine and hypersaline ecosystems, as the resulting ammonium is highly toxic under these conditions. Moreover, this pathway is hardly available in DJP depleted with animal organics which is the main natural source of urea. This is likely why our halophilic strain DJP025-4 lacks ureolytic activity as demonstrated by the phenol red assay and does not contain urease gene in its genome. However, genome analysis revealed the existence of different biocalcification pathway. Like all other aerobic heterotrophic prokaryotes, *Vb. flavescens* DJP025-4 cells produced CO_2_ through metabolic activity and respiration. Carbon dioxide is then hydrated by a complex array of carbonic anhydrases (CAs): one β-class CA (VBD025_00620) and two γ-class CA (VBD025_05090 and VBD025_09020). One of the resulting products, the dissociated carbonic acid ion (HCO_3_^−^), triggers the precipitation of Ca^2+^ as calcite or vaterite on the cell wall. Calcium accumulation there is explained by the presence in the cell wall of anionic peptidoglycans and extracellular polymeric substances (EPS), containing teichoic, lipoteichoic, N-acetylmuramic, and hexuronic acids, respectively. They provide negatively charged functional groups that capture free Ca^2+^ ions from the aqueous environment and serve as localized nucleation sites for the precipitation of calcium carbonate crystals (Ebrahimpour Bozorg et al., 2026). The complete set of genes required for the synthesis of these anionic cell wall components is present in isolate 025–4.

It has recently been shown that CA acts as a universal catalyst for bicarbonate/carbonate formation even at low water activity or high chaotrope (Mg^2+^/Ca^2+^) concentrations in two different coastal sabkhas of Qatar (Abdelsamad et al., 2022; Kimbrel et al., 2018). Moreover, it has been shown that mineralization there not only protects cells from stress (hypersalinity and chaotropicity) but also promotes long-term cell survival through encapsulation in extracellular mineral structures, a process termed as “biomineral protection” (Simon et al., 2022). Thus, calcification and carbonic anhydrase activity are crucial adaptive survival mechanisms for *Virgibacillus* species in high-salinity, chaotic environments, such as Don Juan Pond and gypsum-rich ecosystems in the Middle East or gypsum caves in Spain. These processes not only ensure metabolic resistance to environmental stressors by forming a mineral barrier on the cellular/extracellular matrix but also ensure the long-term preservation of the microbiota.

### Genome methylation and calcium-specific stress response changes in methylation patterns

DNA methylation was investigated as a potential component of the response of *Vb. flavescens* DJP025-4 to environmental stress. In addition to its well-established role in bacterial restriction-modification (R-M) systems, condition-dependent DNA methylation may influence gene regulation and provide a potentially reversible mechanism of environmental adaptation without changes in the underlying DNA sequence (Sánchez-Romero and Casadesús, 2020; Smedile et al., 2025). We therefore examined whether Ca-induced chaotropic stress was accompanied by changes in genome methylation patterns.

Methylated nucleotides identified using SMRT Link ipdSummary were analyzed with MotifMaker, revealing three semi-conserved motifs methylated on one strand: CAGTN*a*G, G*c*AGCH and *c*YCANYAD, where lowercase italicized letters indicate methylated residues. Their asymmetric recognition sequences and single-strand methylation are characteristic of Type III MTases, which commonly participate in R-M systems protecting bacteria against foreign DNA (Rao et al., 2014). Three potentially R-M-associated loci were identified: VBD025_00760–VBD025_00765, encoding Type ISP restriction/modification enzymes; VBD025_02820–VBD025_02825, encoding a cytosine MTase and restriction endonuclease; and VBD025_15915, encoding a solitary restriction endonuclease.

SeqWord Motif Mapper (Lefebvre et al., 2025) was used to assess methylation across coding, non-coding and promoter regions. Plots of methylation scores against local sequencing coverage (Figures 8A,B) distinguished two patterns. CAGTN*a*G showed a “canonical” pattern, with high methylation scores increasing with sequencing coverage, characteristic of persistent methylation of recognition sites. The cytosine-containing motifs showed “non-canonical” methylation, with lower scores and maximum methylation around the average sequencing coverage, consistent with conditional methylation occurring in a fraction of the bacterial population (Korotetskiy et al., 2025; Lefebvre et al., 2025).

**Figure 8:**
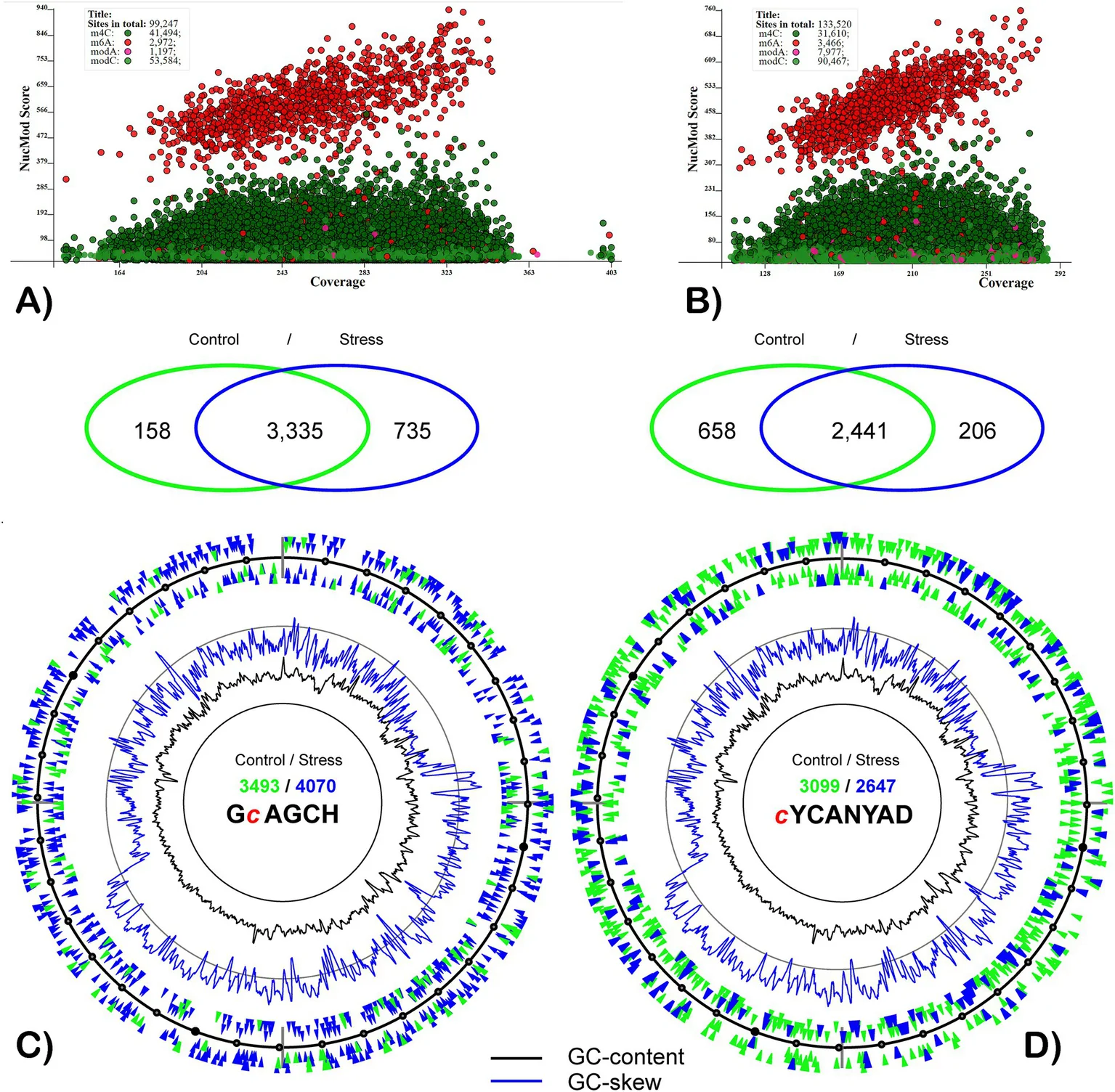
Graphical representation of the distribution of methylated nucleotides. **(A,B)** Dot plots of methylated sites according to local sequencing coverage (X-axis) and methylation prediction scores (Y-axis), constructed for non-stressed and Ca-induced chaotropic stress conditions, respectively. Each dot represents an individual methylated site, with adenine and cytosine residues shown in different colors as indicated in the legend. **(C,D)** Chromosomal distribution of unique, non-shared cytosine-methylated sites detected under normal and chaotropic stress conditions along the *Vb. flavescens* DJP025-4 chromosome, shown for G*c*AGCH and *c*YCANYAD motifs, respectively. Locations of motifs on the chromosome are depicted by triangle marks of green color for the control condition and blue color for the stressed condition. Venn diagrams on the top of circular graphs **(C,D)** show numbers of shared and unique locations of methylated motifs at the control and the osmotic stress conditions.

Adenine methylation was essentially unaffected by growth conditions: 1,123 of 1,129 CAGTN*a*G sites were methylated under both conditions, with six sites consistently unmethylated. Their genomic distribution was close to random, except for a small excess in coding sequences (Table 4). In contrast, cytosine methylation changed substantially under Ca-induced chaotropic stress. Methylation of G*c*AGCH sites increased from 57 to 66%, whereas methylation of *c*YCANYAD sites decreased from 67 to 57% (Figures 8C,D). Under non-stressed conditions, methylated G*c*AGCH motifs were enriched in coding sequences, whereas *c*YCANYAD motifs were enriched in non-coding regions; both were underrepresented in promoter regions. These distribution biases became somewhat less pronounced under stress (Table 4).

**Table 4:** Z-score deviations in the frequencies of methylated sites across coding, promoter, and non-coding regions of the *Vb. flavescens* DJP025-4 genome under different growth conditions.

Motif	Total available	Non-stressed condition				Ca chaotropic stress			
		Total found	Coding	Promoter	Non-coding	Total found	Coding	Promoter	Non-coding
CAGTNaG	1,129	1,123	1.96 ± 0.05; *p* = 0.05	−0.60 ± 0.17; *p* = 0.55	−1.58 ± 0.04; *p* = 0.115	1,123	1.96 ± 0.05; *p* = 0.05	−0.60 ± 0.17; *p* = 0.55	−1.58 ± 0.04; *p* = 0.115
GcAGCH	6,170	3,493	7.39 ± 0.03; *p* = 0.0	−7.48 ± 0.10; *p* = 0.0	−4.69 ± 0.02; *p* = 0.0	4,070	6.70 ± 0.02; *p* = 0.0	−8.09 ± 0.09; *p* = 0.0	−3.93 ± 0.02; *p* = 0.0
cYCANYAD	4,620	3,099	−13.07 ± 0.03; *p* = 0.0	−6.27 ± 0.10; *p* = 0.0	12.98 ± 0.02; *p* = 0.0	2,647	−11.85 ± 0.03; *p* = 0.0	−5.77 ± 0.11; *p* = 0.0	11.79 ± 0.03; *p* = 0.0

The different responses of adenine and cytosine methylation provide a possible biological link between DNA methylation and adaptation to Ca-induced chaotropic stress. Adenine methylation remained essentially constant, consistent with a constitutively active R-M system, whereas both cytosine methylation patterns were condition-dependent (Figure 8). Because DNA methylation can influence bacterial gene regulation, these changes may represent an additional regulatory component of the stress response. However, the data demonstrate an association between Ca stress and altered methylation rather than a causal effect on transcription. Interestingly, only one cytosine-specific MTase (VBD025_02820) was annotated, suggesting either an unidentified additional MTase or recognition of both motifs by the same enzyme with condition-dependent specificity.

### Calcium-specific transcriptomic stress response of DJP025-4

Volcano and M-plots showing differential gene expression under Ca^2+^-induced chaotropic stress are presented in Figure 9. Only differentially expressed genes with false discovery rate (FDR) ≤ 0.05 were considered. A total of 2,330 genes were differentially expressed, showing a balanced distribution of 1,185 upregulated and 1,145 downregulated DEGs (Supplementary Table 2). Initial analysis of the upregulated genes expression indicate that they were predominantly associated with transport functions, osmoadaptation, and stress response mechanisms, while downregulated genes were primarily involved in translation, biosynthesis, and central metabolism. This coordinated regulation indicates a shift from growth to survival, defining a conserved physiological strategy under CaCl_2_-induced stress, the feature which was observed by SEM imaging.

**Figure 9:**
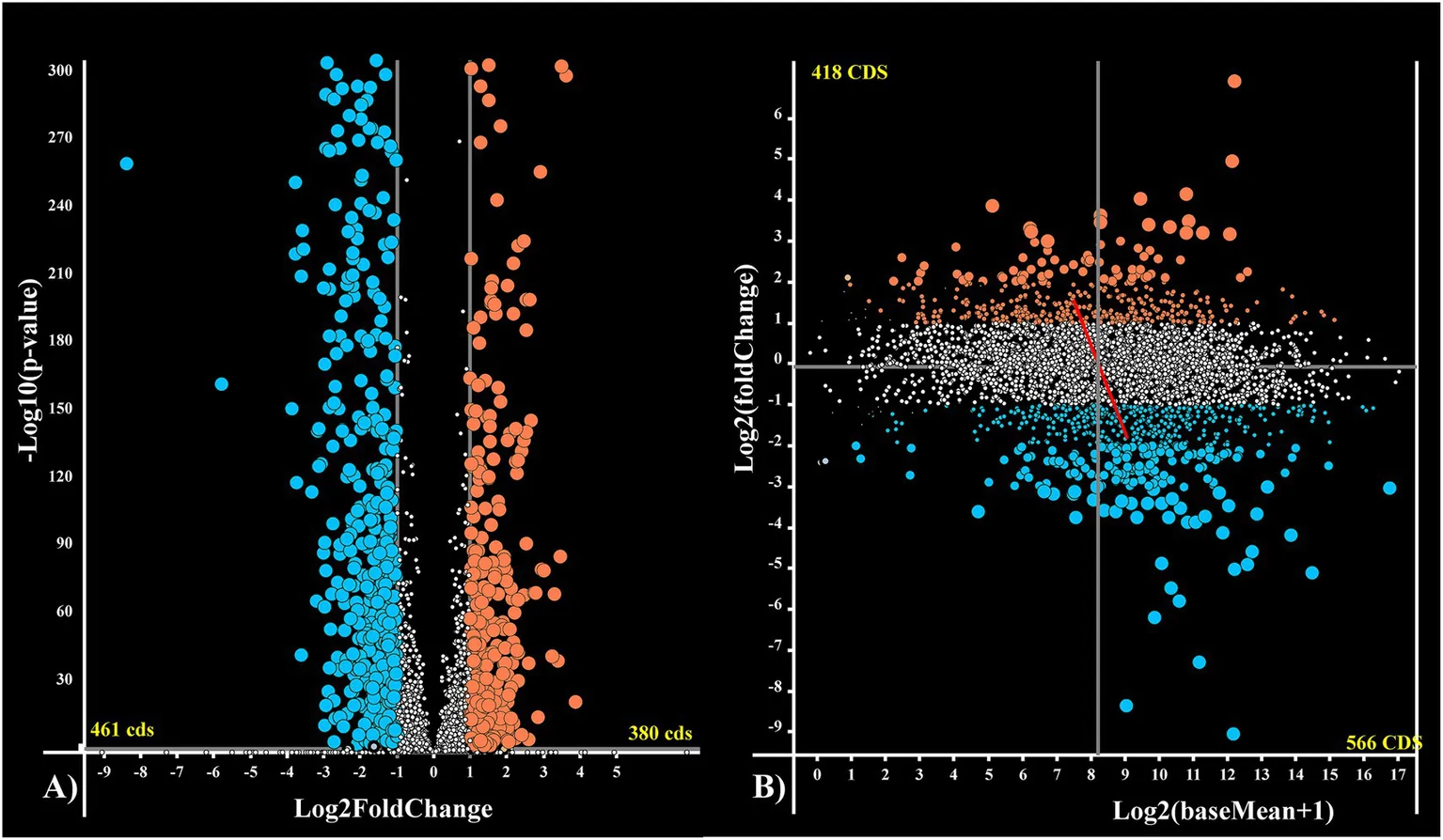
Differential gene expression in *Vb. flavescens* DJP025-4 induced by chaotropic stress. **(A)** Volcano plot showing differential gene expression according to Log_2_(fold change) and −log10(*p*-value). **(B)** M-plot showing differential gene expression according to Log2(BaseMean + 1) and Log_2_(fold change). Each gene is represented by a dot, with red and blue indicating up- and down-regulated genes, respectively. Larger dots represent genes with absolute fold-change values greater than four. White dots represent genes that were not differentially expressed. The red trend line shows predominance of up- or down-regulation among highly expressed genes.

Primarily we focused on genes involved in compatible solute(s) metabolism and transport, potassium homeostasis, mechanosensitive channels, and cation/proton antiporters. Glycine betaine/choline operon, comprising betA (VBD025_04585), betB/gbsA (VBD025_04590), a ProX/OpuAC-like substrate-binding protein (VBD025_04595), and the transcriptional regulator gbsR (VBD025_04600), was significantly upregulated in MBSW70 compared to that in MBSW70 + 1 M. Thus, the transformation of choline to glycine betaine was significantly repressed under CaCl_2_ stress. In contrast, at least four different types of high-affinity osmoprotectant transport systems were significantly upregulated under calcium-supplemented conditions, indicating selective activation of the uptake of exogenous compatible solutes by DJP025-4 cells in response to CaCl_2_. These systems include (i) the ProU (proVWX) system (VBD025_17720–30), a high-affinity ABC transporter of glycine betaine and proline into the cell; (ii) the OpuA (osmoprotectant uptake) system, consisting of OpuAC operon (VBD025_11930–5) and OpuB (VBD025_01155); and (iii) the multicomponent ABC transporter BusA (betaine uptake system) VBD025_17445–65. It should be noted that in Gram-positive bacteria, the Bus system exhibits the opposite behavior (repression versus induction), regulated by external osmolality and the c-di-AMP-sensitive transcriptional regulator BusR (Li et al., 2026). When cells are exposed to high salt levels or osmotic stress, the Bus system is induced to transport extracellular betaine, but high intracellular betaine levels activate BusR expression, which suppresses the system’s activity. The BusR transcriptional regulator VBD025_17470 is located adjacent to the BusA operon in the DJP025-4 genome, and its transcription was repressed in the presence of calcium (Supplementary Table 2). Finally, (iv) an osmoprotectant transport system, VBD025_13205, belonging to the single-component OpuD/BelL system of the BCCT (betaine-carnitine-choline transporter) family of transporters, also responded positively to calcium addition. Expression of the KtrC protein (VBD025_13255), responsible for potassium uptake, was also induced in the presence of CaCl_2_; this indicates the activation of ion and osmotic balance mechanisms and aligns with the role of K^+^ homeostasis in the osmoadaptive response known as the “salt-in” strategy (accumulation of potassium salts within the cell). We specifically analyzed intracellular K^+^ concentration and accumulation under CaCl_2_-induced stress using energy-dispersive spectroscopy (EDS) (Figure 4; Supplementary Figures S1–S7). Indeed, some elemental distribution maps clearly demonstrate an association between potassium and the cells (Figure 4). However, repeated application of this method revealed low reproducibility for this association. In the halite control sample, co-precipitation of K^+^ with NaCl was observed during sample preparation (Supplementary Figures S1–S3), whereas in some samples (Supplementary Figures S4–S7), the potassium distribution differed and showed weak correlation with both the cells and the NaCl crystals. Nevertheless, in all areas, the potassium content in the cellular regions slightly exceeded background levels, which indicates that *Vb. flavescens* also employs an ion-balance mechanism characteristic of haloarchaea (the “salt-in” strategy) to cope with elevated calcium concentrations, albeit to a minor extent. At the same time, CaCl_2_ stress was associated with repression of the Mrp/Mnh Na^+^/H^+^ antiporter complex. Subunits mnhA, mnhD, mnhE, mnhF, mnhG (VBD025_10265–90), and mnhB (VBD025_12000) all showed significantly lower expression in MBSW70 + 1 M than in MBSW70. This suggests that the response to CaCl_2_ was not driven by induction of the multicomponent Na^+^/H^+^ antiporter system, but rather by selective osmoprotectant uptake and potassium homeostasis.

Of particular interest in terms of providing additional sites for calcite crystallization on the cell surface is the increased expression of several cell appendages genes: these are the genes of the flagellar proteins FlaA (VBD025_07600; K02406) and FliT (VBD025_07885; K02423), which are known to serve as an H-antigen on the cell surface and ensure the inclusion of hook-associated axial proteins, respectively (Aizawa and Minamino, 2024; Bennett et al., 2001). Surface structures associated with these proteins can additionally serve as crystallization centers for calcite. Three more DEGs in DJP025-4 related to cell appendages encode the tight pili adherence system (VBD025_08550–60), consisting of TadB and TadC proteins, and the pili dynamics ATPase TadA (CpaF), which controls the elongation and contraction cycles of pili, responsible for cell adhesion and colonization (Hohl et al., 2024). In addition to the genes mentioned, highly positive DEGs were involved in general catabolic processes, such as respiration (the gene VBD025_13260, encoding the cytochrome *bd* ubiquinol oxidase subunit II), the tricarboxylic acid cycle (*sucB* gene of 2-oxoglutarate dehydrogenase complex, VBD025_17795), terminal heme-copper oxidoreductase biosynthesis (P-type Cu^2+^ transporter *copB* gene, VBD025_16150), and the *spoIIQ* gene (VBD025_05925), which is required for anchoring membrane proteins on the forespore side of the septal membrane in bacilli (Campo et al., 2008). Additional evidences on modification of DJP025-4 cell surface under the Ca^2+^ stress can also be provided by the detection of overexpression (logFC ≥ 2) of multiple enoyl-CoA hydratases (VBD025_05125–3 and VBD025_05145), known to be part of the fatty acid signal biosynthesis and biofilm development machinery (Cameron et al. 2019; Mitchell and Ellermann, 2022). We also found strong positive response to Ca^2+^ stress for at least two genes, VBD025_06695 and VBD025_07560, of a beta-lactamase class C family protein (UniProt entry A0A7W5YR43), which possess peptidase activity and a penicillin-binding site, thus being a putative determinant of DJP025-4 cell surface protein and peptidoglycans modification, along with several other putative CaCl_2_-positive peptidases (Supplementary Table S2). Cell wall modification as a response to Ca^2+^ stress was also supported by overexpression of glycan-modifying enzymes, such as beta-N-acetylglucosaminidase. Among the five such enzymes harbored in the DJP025-4 genome, at least two, VBD025_0385 and VBD025_07555, have a logFC ≥ 2.

In general, in addition to the osmoadaptation strategies discussed above, transcriptomic analysis revealed that the basic metabolic response of strain DJP025-4 to increase of Ca^2+^ content in the medium preceding calcite formation is the intensification of cell surface structure biosynthesis, including peptidoglycan metabolism, biofilm matrix formation, and cellular appendages assembling. The most pronounced expression response of some regulatory genes and transporters to the presence of calcium may be associated with a switch in metabolic processes toward salt resilience and calcite precipitation as one of the indirect mechanisms of salt resistance. Consequently, cell surface modification with additional polypeptides, peptidoglycans, sugar prosthetic groups of cell surface proteins, or cell appendages could significantly increase the amount and spatial distribution of potential calcite crystallization sites on the cell surface providing the organism for high calcinating activity, as confirmed microscopically and by EDS analysis (Figure 4).

## Conclusion

This study investigated ephemeral bubble-containing microbial mats on the southern periphery of DJP, where CaCl_2_-rich subsurface brines mixed with meltwater from a nearby glacier. Attempts to culture calcium-resilient bacteria resulted in the recovery of several isolates belonging to the phyla *Bacillota* and *Pseudomonadota*. Experimental testing of the most calcium-tolerant isolate, *Virgibacillus flavescens* DJP025-4, revealed its remarkable resistance to varying concentrations of CaCl_2_ and perchlorate brines. Microscopic examination (SEM and cryo-ET) and EDS analysis confirmed that the strain possesses strong biocalcification activity, which leads to the formation of a hard calcite shield on the outer surface of cells, thereby protecting them from calcium toxicity by hindering its penetration into the cells. Biocalcification in the organism is not related to urease activity thus providing an example of adaptation to both salt and low organics stresses. Transcriptomic analysis revealed that the primary metabolic responses of strain DJP025-4 to elevated Ca^2+^ levels include increased uptake of compatible solutes, cell surface modification, biofilm matrix formation, and cellular appendages assembling increasing the cell surface area and number of possible calcite nucleation sites. The fact that DJP025-4 cells, in the presence of calcium, are oriented toward the uptake of external compatible solutes rather than *de novo* synthesis of glycine betaine indicates their dependence on other members of the microbial consortium that produce solutes. Thus, these rarely observed DJP microbial communities may not only survive but also function in this “Martian” chaotropic ecosystem solely through the tight interaction of a trophic and interdependent network. These data enabled us to propose a model for the functioning of strain DJP025-4 in the calcium-saturated brines of the DJP stream—conditions incompatible with the survival of common microorganisms. This model parallels the ecosystem dynamics of the hypersaline deep-sea Medee Lake, which relies on glycine betaine (GB) produced in overlying layers (Yakimov et al., 2013). In the stratified ecosystem of the DJP stream, microbial mats developing at the surface, specifically, in a lower-salinity layer situated above the main brine body of the stream, are characterized by high microbial biomass density; this biomass evidently consists of moderate halophiles that synthesize osmolytes (a “salt-out” adaptation strategy). We believe that this production activity provides strain DJP025-4 with its unique ability to function in deeper horizons: rather than synthesizing them itself, the strain consumes the energy-expensive osmolytes produced in the overlying layers.

Whole-genome sequencing of *Vb. flavescens* DJP025-4 provided insights into genome-wide adaptive changes that enable this extremophile to withstand the harsh conditions of Don Juan Pond ecosystem, including extreme UV irradiation, low temperatures, and extremely high concentrations of calcium ions. Multiple loci containing central metabolic genes but characterized by atypical tetranucleotide abundance patterns suggest that natural genetic transformation may contribute to the adaptation of this bacterial community to rapidly changing environmental conditions. The seasonal influx of meltwater reduces the chaotropicity of the environment and creates a temporary window for bacterial growth and proliferation, necessitating a rapid transcriptional transition from a homeostasis-maintenance regime to active metabolism. The stress-dependent changes in cytosine methylation observed in DJP025-4 suggest that DNA methylation may represent an additional regulatory component of its response to rapidly changing environmental conditions. Whether these methylation changes directly contribute to the observed transcriptional response remains to be experimentally established. While no prophage insertions were identified in the sequenced genome, the presence of an active restriction-modification system resulting in persistent methylation of adenine residues at CAGTNaG motifs suggests continuing selective pressure from invasive bacteriophages, even in this harsh environment resembling Martian conditions.

## Data Availability

The datasets presented in this study can be found in online repositories as well as in Supplementary Tables 1, 2. The obtained 16S rRNA gene sequences were deposited in the NCBI GenBank database; they were assigned accession numbers PZ879541- PZ879543 (for isolates DJP025-1, DJP025-2 and DJP025-3, respectively). The 16S rRNA gene sequence of isolate DJP025-4 was assigned accession number QB082405. The barcoding sequencing dataset used in this study and related to ephemeral microbial mats of the DJP025-4 is freely available through the European Nucleotide Archive (ENA)/NCBI under the accession number: PRJNA1495185. The assembled genome of *Virgibacillus flavescens* DJP025-4 was registered on NCBI GenBank under BioProject PRJNA1265159, BioSample SAMN48586054, and accession number CP192260. RNA-Seq experiments was registered on NCBI SRA under same BioProject and BioSample, with accession numbers SRR39660586 (D025_4_MB_A_1), SRR39663298 (D025_4_MB_B_1), SRR39663297 (D025_4_MB_C_1), SRR39660589 (D025_4_1M_A_1), SRR39660588 (D025_4_1M_B_1), SRR39660587 (D025_4_1M_C_1). PacBio Hi-Fi raw reads of *Virgibacillus flavescens* DJP025-4 genome can be accessed via link https://www.ncbi.nlm.nih.gov/sra/SRX35040965[accn].

## Footnotes

1
https://www.macrogen-europe.com

2
https://www.arb-silva.de/documentation/release-138/

3
https://zenodo.org/record/4587955#.YgKJlb_MJH4

4
https://www.kbase.us/

5
https://www.geneious.com

6
https://github.com/chrilef/BactEpiGenPro

7
https://github.com/SeqWord/GISniffer.3.1

8
http://folk.uio.no/ohammer/past/
