# Supplementary material for: Mechanisms of unique chaotropic resistance and biocalcification in a halophilic Virgibacillus isolated from ephemeral microbial mats of the calcium-rich Don Juan pond in Antarctica
Source: Front Microbiol. 2026 Oct 28;17:1980805. doi: 10.3389/fmicb.2026.1980805 (PMC13753959; doi:10.3389/fmicb.2026.1980805)
Supplement: Supplementary file 1 [file Data_Sheet_1.pdf]

SUPPLEMENTARY INFORMATION

Laura Marturano et al.

Mechanisms of unique chaotropic resistance and biocalcification in a halophilic *Virgibacillus* isolated from ephemeral microbial mats of the calcium-rich Don Juan Pond in Antarctica.

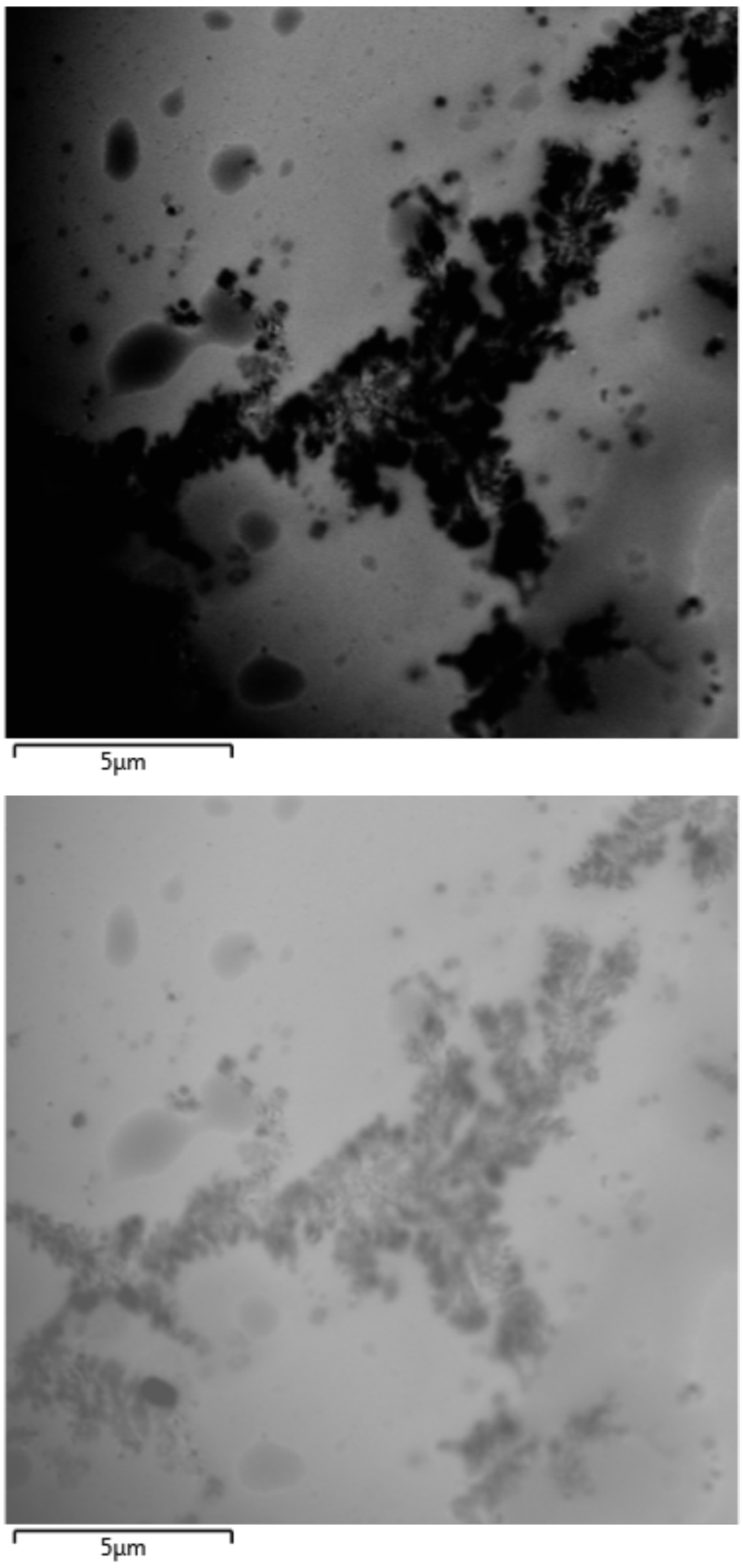

Supplementary Figure S1. Control spot (spot 2) with NaCl crystals precipitated during the sample preparation

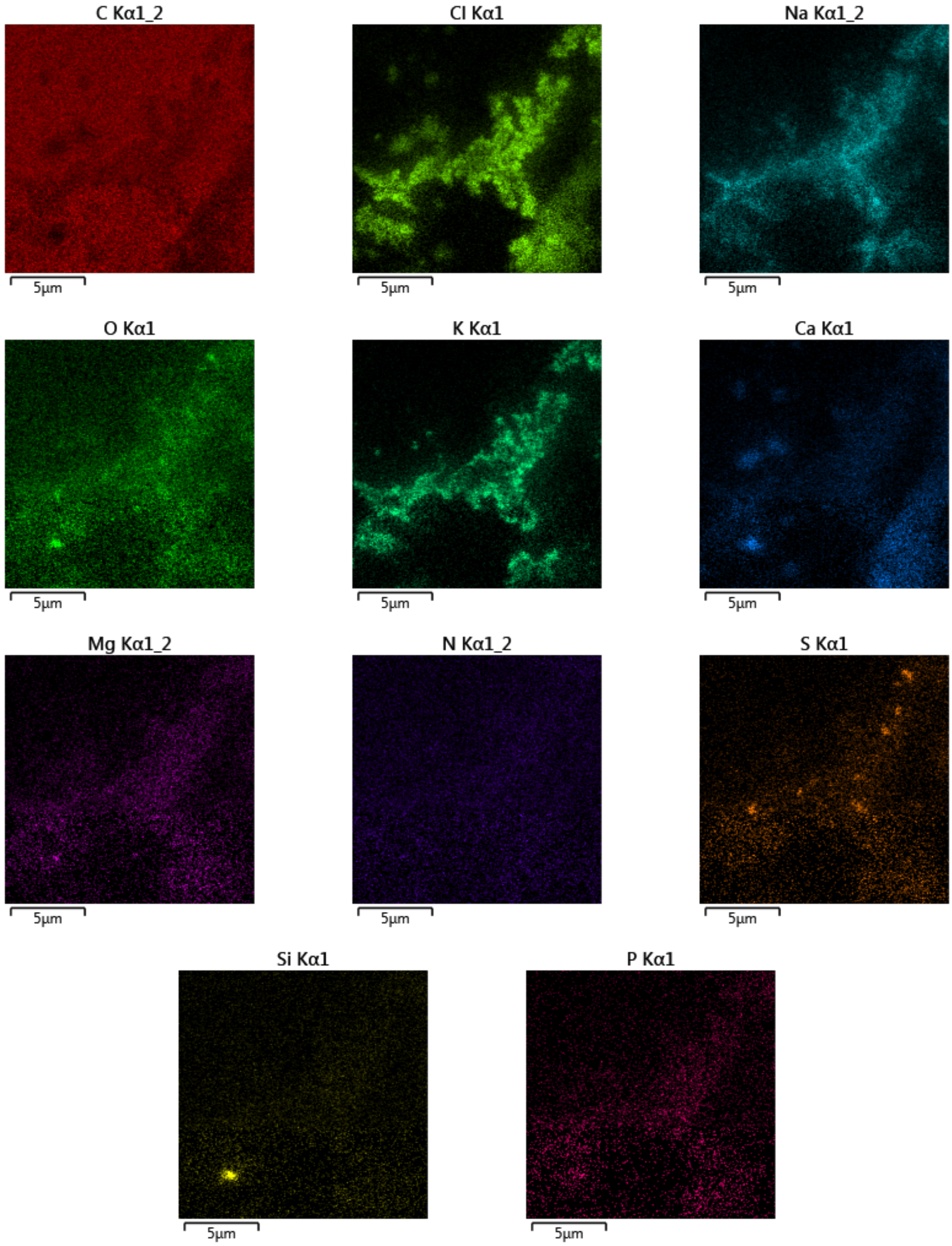

Supplementary Figure S2. Elemental map of the same NaCl aggregate depicted in Figure S1. Note clearly visualized Na(K)Cl crystals, extremely low content of Ca, Si and N.

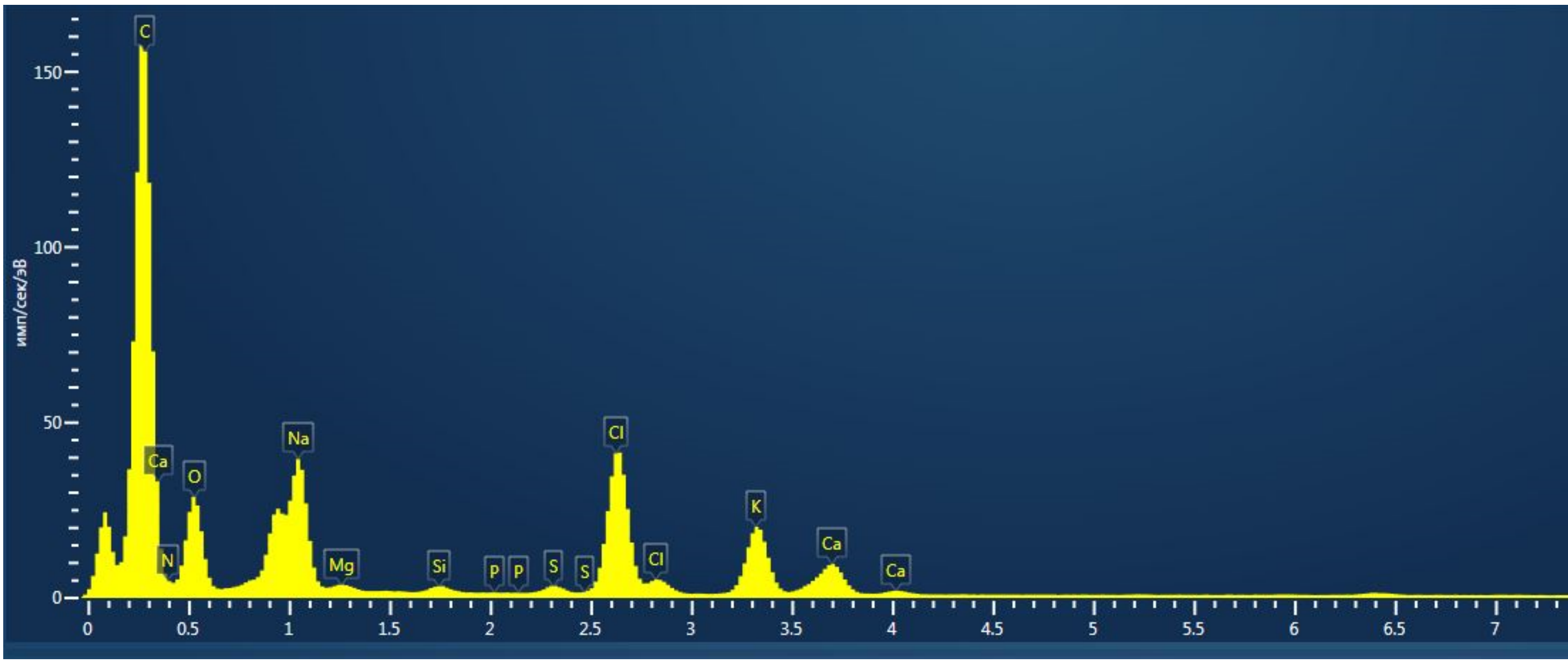

Supplementary Figure S3. Summarized spectrum of the elemental map of NaCl aggregate depicted in Figure S1.

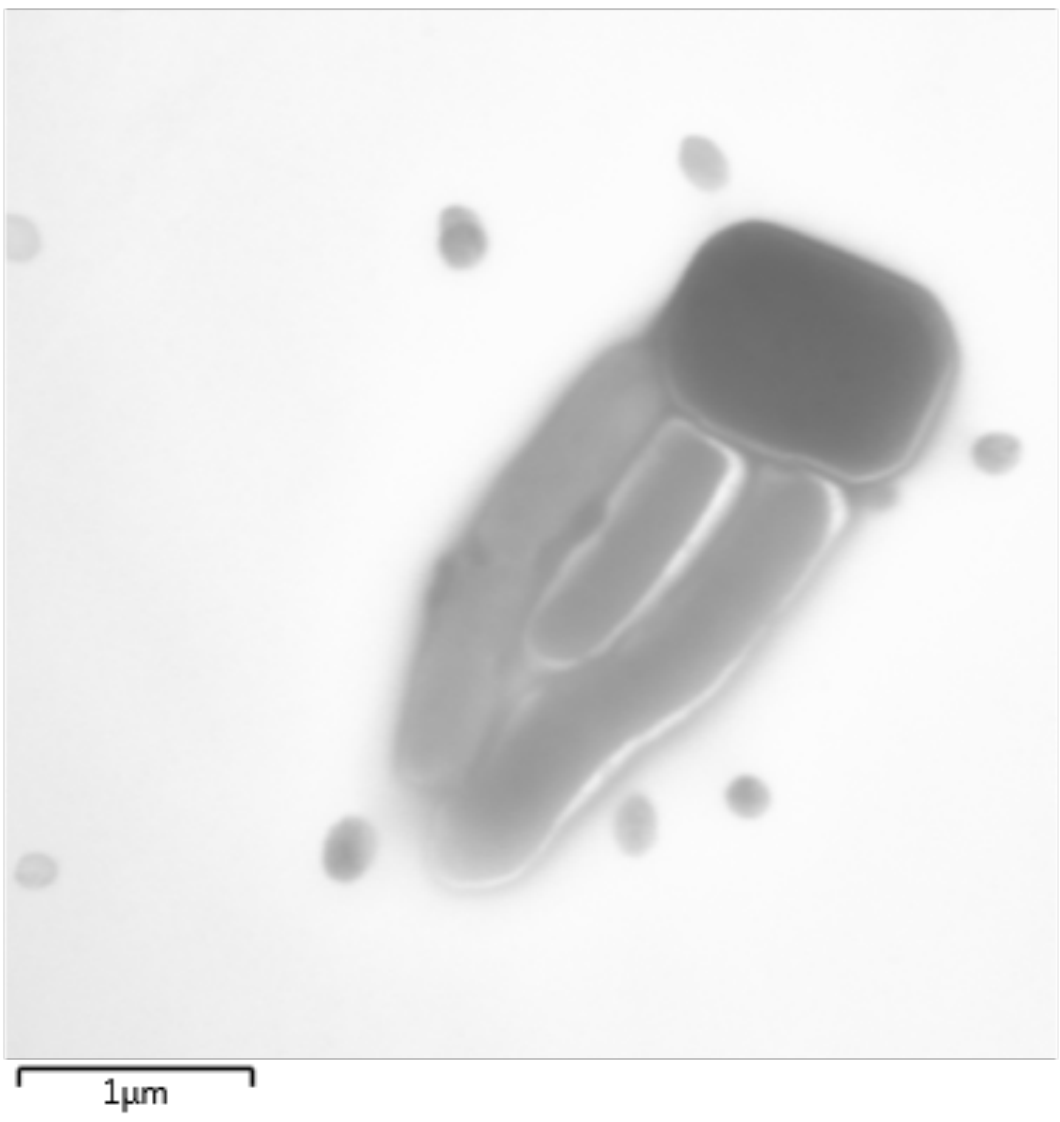

Spot 3

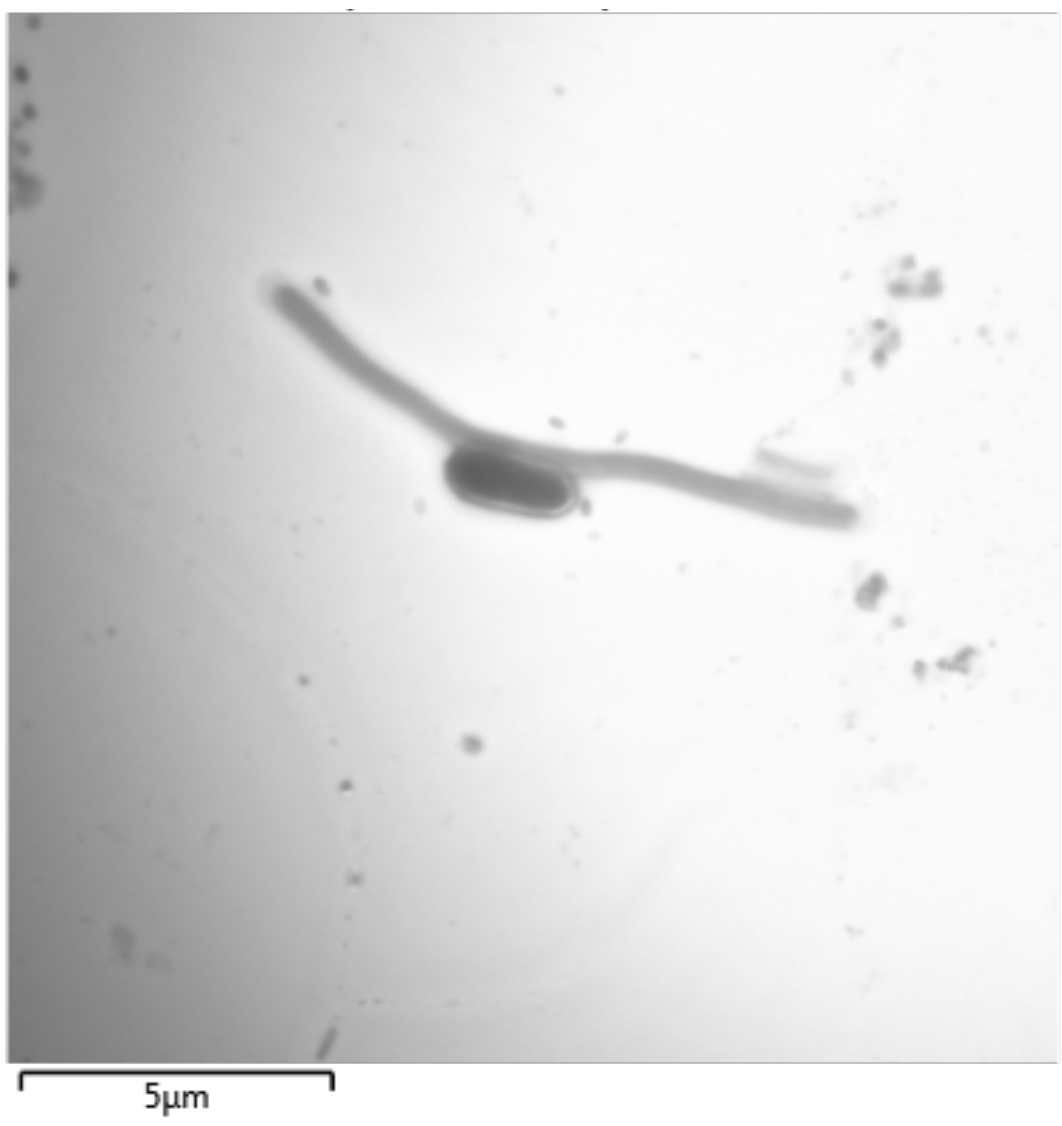

Spot 4

Supplementary Figure S4. Culture of *Vb. flavescens* DJP025-4 grown on MBSW70+1M medium (supplemented with 1 M $CaCl_2$); spots 3 and 4. Biocementation ($CaCO_3$ formation) is clearly visible as white films on the cell surfaces, alongside extracellular NaCl crystals appearing as dense, dark, rectangular or oval formations.

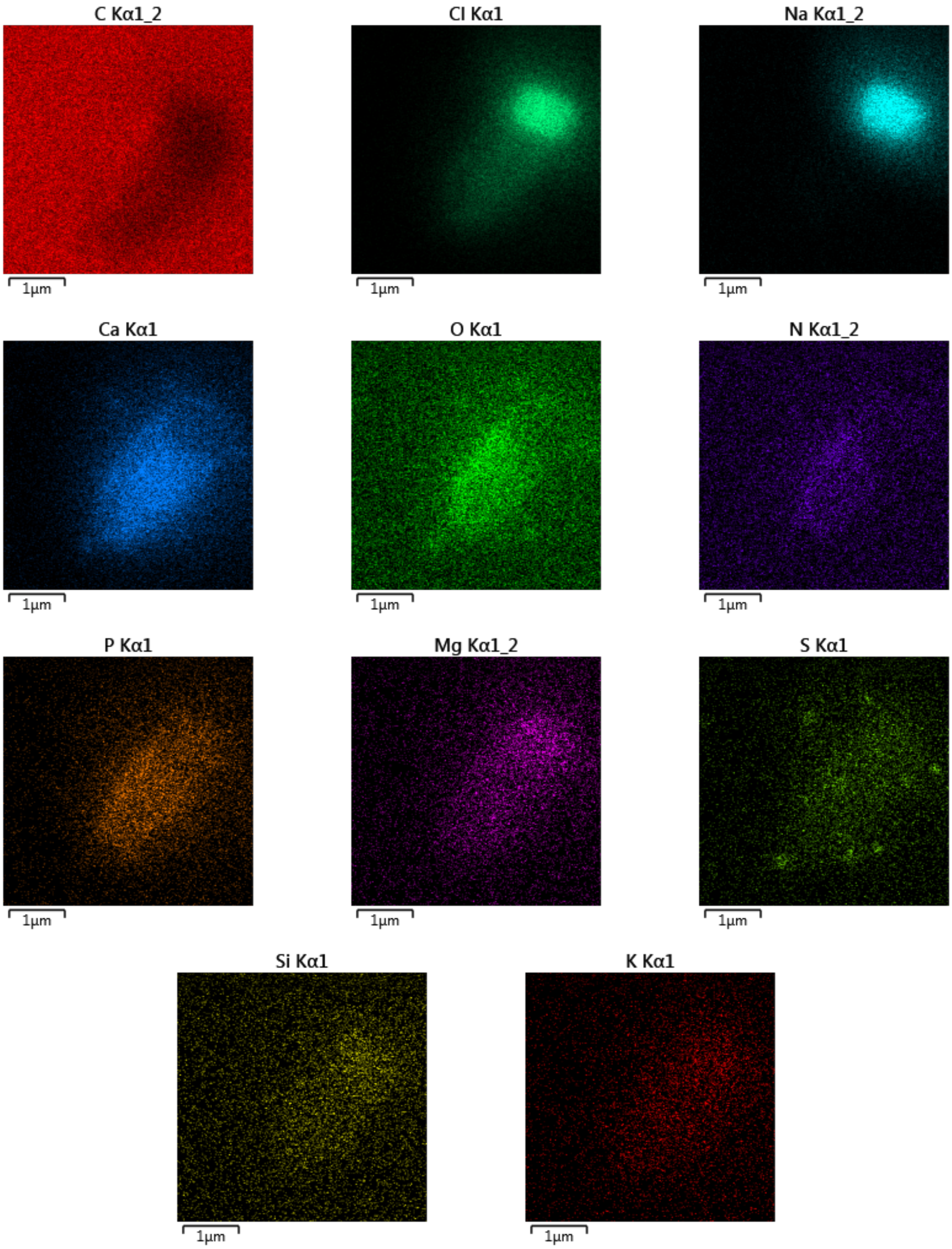

Supplementary Figure S5. Elemental distribution map for the spot 3. Note the clear separation of the NaCl crystal from the cells, as well as the precise overlap of the distribution areas for Ca, O, N, Mg, P (and, to a lesser extent, S) with the cell locations; this contrasts with the distribution patterns of potassium and Si.

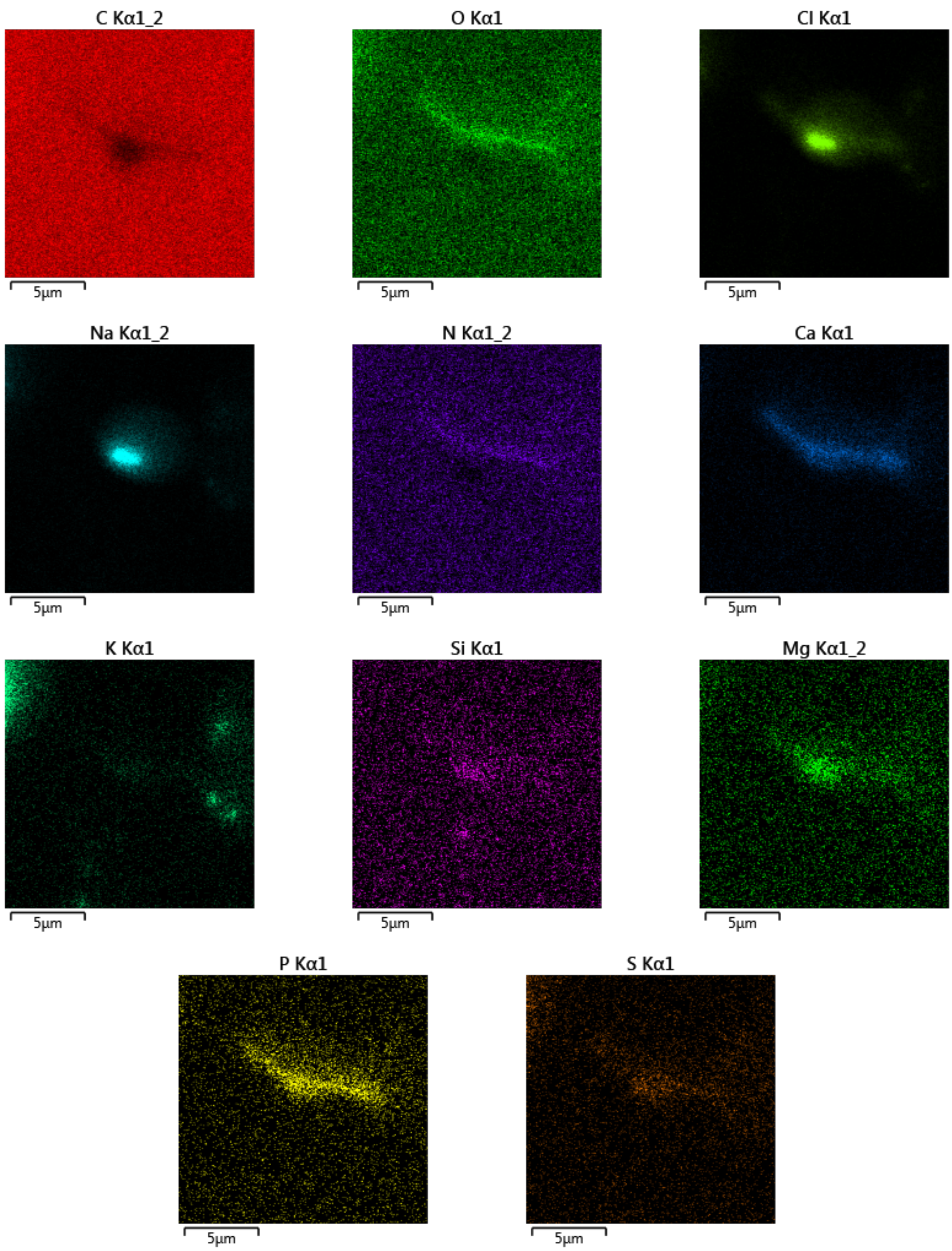

Supplementary Figure S6. Elemental distribution map for the spot 4. Note the clear separation of the NaCl crystal from the cells, as well as the precise overlap of the distribution areas for Ca, O, N, Mg, P (and, to a lesser extent, S and Si) with the cell locations; this contrasts with the distribution patterns of potassium.

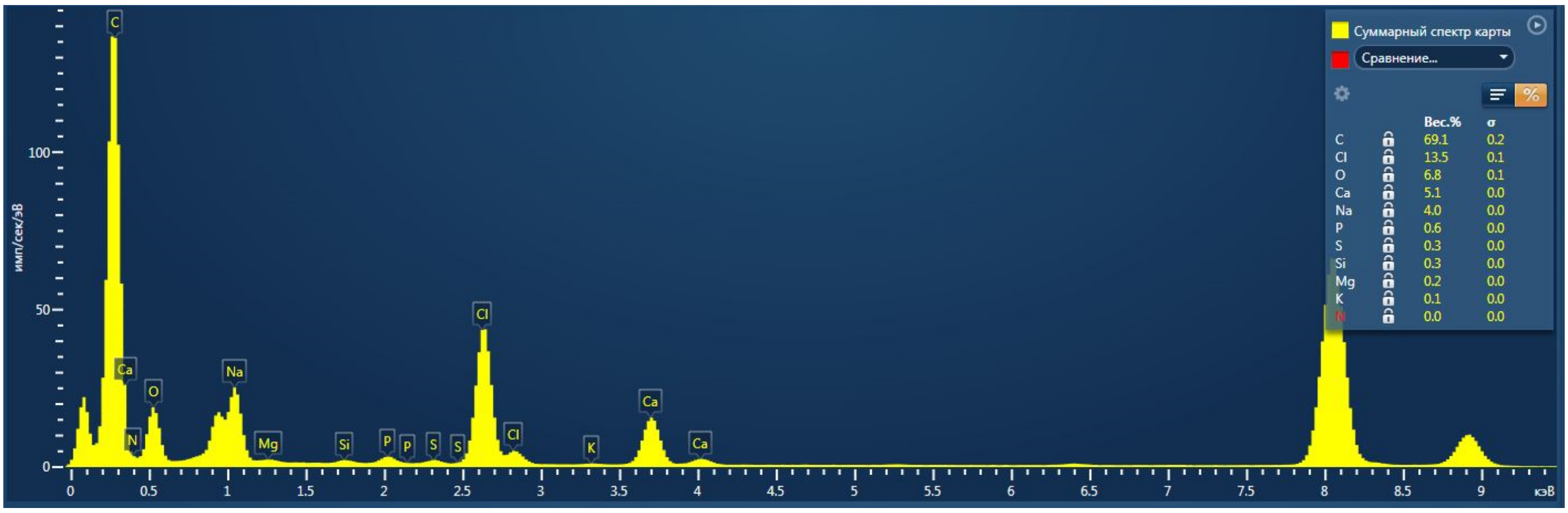

Spot 3

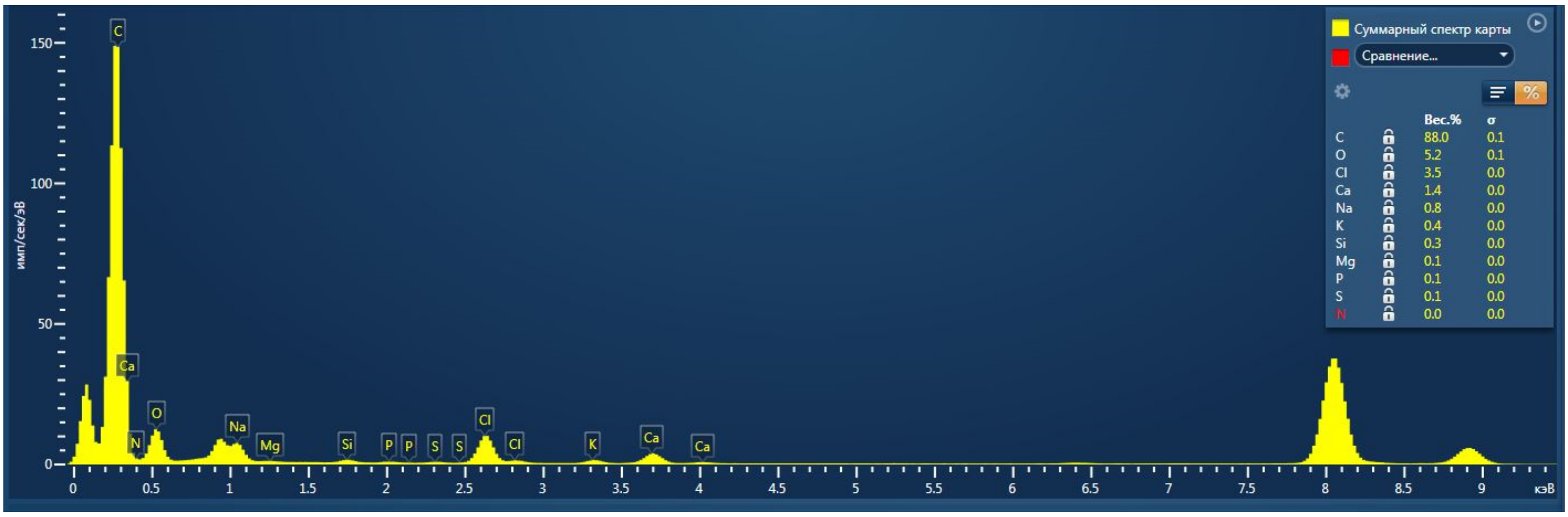

Spot 4

Supplementary Figure S7. Summarized spectrum of the elemental maps of spot 3 and spot 4 depicted in Figure S4.

Conclusions:

Elemental maps of spot 3 clearly show potassium association with the cells. However, this association is poorly reproducible with the applied technique. The control spot 2 shows K co-precipitation with NaCl upon sample preparation, but spot 4 demonstrates different potassium distribution along the samples which poorly correlates both with the cells and NaCl crystals. Still, at all the spots with the cell's potassium content is higher on the cell images than at the background. More pronounced effect is observed with Mg, which reveals stronger correlation with the cells and is almost completely absent at the spots with NaCl crystals, both in the culture samples (spots 1, 3, 4) and in the control (spot 2) (Figure 4 and Supplementary Figures S1-7).
